# Exposure Prioritization (*Ex Priori*): A Screening-Level High-Throughput Chemical Prioritization Tool

**DOI:** 10.3390/toxics10100569

**Published:** 2022-09-28

**Authors:** Heidi F. Hubbard, Caroline L. Ring, Tao Hong, Cara C. Henning, Daniel A. Vallero, Peter P. Egeghy, Michael-Rock Goldsmith

**Affiliations:** 1 ICF International, 2635 Meridian Parkway, Durham, NC 27713, USA; 2 Chemical Characterization and Exposure Division, Center for Computational Toxicology and Exposure, Office of Research and Development, U.S. Environmental Protection Agency, Durham, NC 27713, USA

**Keywords:** high-throughput exposure modeling, consumer products, consumer habits and practices

## Abstract

To estimate potential chemical risk, tools are needed to prioritize potential exposures for chemicals with minimal data. Consumer product exposures are a key pathway, and variability in consumer use patterns is an important factor. We designed *Ex Priori*, a flexible dashboard-type screening-level exposure model, to rapidly visualize exposure rankings from consumer product use. *Ex Priori* is Excel-based. Currently, it is parameterized for seven routes of exposure for 1108 chemicals present in 228 consumer product types. It includes toxicokinetics considerations to estimate body burden. It includes a simple framework for rapid modeling of broad changes in consumer use patterns by product category. *Ex Priori* rapidly models changes in consumer user patterns during the COVID-19 pandemic and instantly shows resulting changes in chemical exposure rankings by body burden. Sensitivity analysis indicates that the model is sensitive to the air emissions rate of chemicals from products. *Ex Priori*’s simple dashboard facilitates dynamic exploration of the effects of varying consumer product use patterns on prioritization of chemicals based on potential exposures. *Ex Priori* can be a useful modeling and visualization tool to both novice and experienced exposure modelers and complement more computationally intensive population-based exposure models.

## 1. Introduction

In modern society, exposure to a wide range of chemical substances through our daily habits and routines is unavoidable. Indeed, an estimated 8 million chemicals are commercially available [1], with more than 80,000 chemicals regulated under the Toxic Substances Control Act [2]. The likelihood that a substance will cause an adverse health effect depends not only on the chemical’s hazard or toxicity, but also on the exposure or dose level. Recognizing this, the Frank R. Lautenberg Chemical for the 21st Century Act reformed the Toxic Substances Control Act (TSCA) to require the evaluation and regulation of chemical substances based on their human health and ecological risk potential. To meet the requirements of TSCA, evaluation must be based on reliable values for both exposure potential and hazard potential, since risk is a function of both. For many chemicals, much of the risk uncertainty is tied to the paucity of sound exposure information [3]. In the absence of exposure, risk calculations relying on default exposure values become highly uncertain. To support this the U.S. Environmental Protection Agency (EPA)’s Chemical Safety for Sustainability (CSS) research program has been developing new ways to evaluate chemicals used in consumer products and articles. These new methods focus on identifying potentially problematic chemicals before they reach the marketplace by estimating both exposure and hazard potential.

Exposure assessment encompasses both external and internal components. External exposure includes those scenarios in which a substance is released into the environment and reaches a human or ecosystem receptor. With far-field sources, the substance is most often transported through the environment, and sometimes transformed, before reaching the receptor. Often, far-field exposures are estimated and ranked using proxy information, such as the amount of chemical produced per year and chemical properties that might affect bioaccumulation and persistence [4]. However, the principal scenarios for many chemical exposures often do not involve far-field sources. Indeed, many chemical exposures among the general population follow the release of substances from near-field sources, such as household and personal care products, furnishings, and building materials [3,5,6,7,8,9]. Estimating these exposures relies on assumptions about how often products are used (activity patterns and exposure interactions) and which products contain a given chemical [6]. Activity patterns of behavior and product use may vary greatly among individuals, making the ranking of chemical intakes complicated and uncertain [6].

The second important component of exposure is what happens to the chemical after it enters the body. Traditionally, exposure estimates have focused on external dose, or the amount of chemical that is ingested, inhaled, or applied to the skin. However, the potential for toxicity is better represented by internal dose, which accounts for the absorption, distribution, metabolism, and excretion (ADME) of the chemical in the body. For example, for two chemicals with similar modes of action for toxicity, the chemical that is minimally absorbed and completely eliminated in 24 h is less problematic than another chemical which is readily absorbed and slowly eliminated [10,11]. Internal dose is also a useful metric because it may be compared with any existing biomonitoring data for model validation. However, ADME data, and particularly metabolism rates, have not been available for a wide range of chemicals, so estimation of internal dose is difficult.

The uncertainties associated with these components of exposure estimation call for an evaluation method that accounts for chemical-specific differences in ADME processes and allows the user to investigate the effect of different assumptions about use and activity patterns (e.g., “highly-exposed individual”, “general population”, or “occupational user”) on the chemical rankings [9,11,12,13,14]. Accounting for these differences can allow the exposure model to be tailored to individual user’s needs and allow for comparison with toxicity or hazard data.

To this end we have developed *Ex Priori* (abbreviated form of “exposure prioritization”), an Excel-based dashboard-type chemical evaluation tool to estimate and rank chemical exposure potential from a wide array of consumer products across exposure routes based on internal dose. *Ex Priori* is designed to quickly provide a snapshot of exposure potential based on internal dose, accounting for absorption and metabolic biotransformation. Because *Ex Priori* is designed to function with either user-supplied inputs or default model inputs, it can quickly produce rankings that are flexible to changes in model inputs. In this way, *Ex Priori* can be a useful modeling and visualization tool to both novice and experienced exposure modelers. *Ex Priori* complements more computationally intensive population-based exposure models, such as SHEDS-HT, EPA’s stochastic high-throughput exposure model [6]. *Ex Priori* also complements more comprehensive life-cycle exposure assessment models such as USETox [15,16] and RAIDAR [17,18,19,20]. Detailed models such as these require more computational power; more extensive exposure data; and/or a deeper understanding of exposure modeling, product uses, chemicals, use environments, and populations. Because *Ex Priori* differs from traditional exposure screening tools by ranking by internal dose rather than by intake, it can be compared with complementary data about hazard or toxicity (i.e., in vitro high-throughput screening assays such as ToxCast^TM^ [21], or traditional in vivo data, using toxicokinetic modeling to convert applied dose to internal dose), and potentially used to inform risk.

The presented research describes how the *Ex Priori* tool considers various routes of exposure and introduces the inputs, exposure routes, and models used. Currently, the *Ex Priori* tool ranks 1108 chemicals present in 228 separate consumer product categories from highest to lowest exposure potential. As an adaptable systems framework, *Ex Priori* synthesizes knowledge from various domains and has the ability to add more chemicals and products as information becomes available. A one-way sensitivity analysis explores the most uncertain model inputs. Finally, the findings of this study and implications for future research directions are discussed.

## 2. Materials and Methods

**Figure 1:**
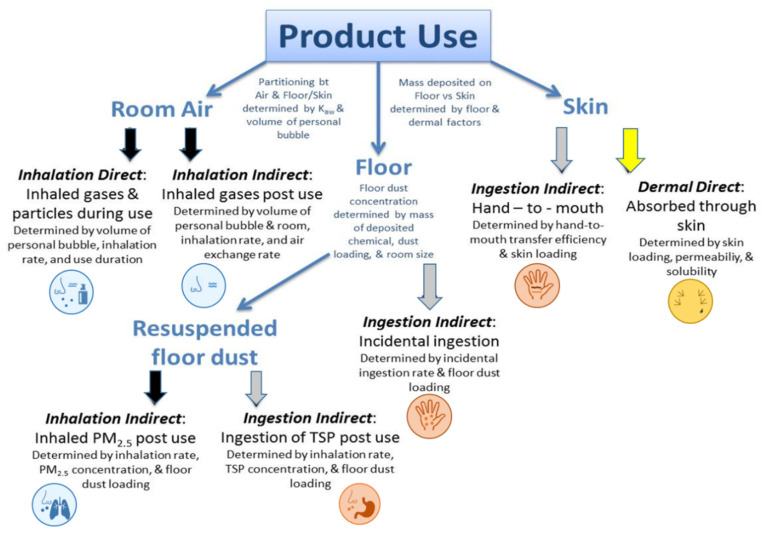
Apportionment of mass by exposure route in *Ex Priori*.

*Ex Priori* is intended to prioritize exposure potential of chemicals released into the indoor environment from consumer product use. *Ex Priori* deterministically models potential exposures. To do this, *Ex Priori* uses product-specific data (Table S1), whereby product composition data (mass fraction as grams chemical per gram product) are combined with consumer habits and practices data (i.e., frequency of use for each consumer product, the amount of product used each time, and the duration of each product use). Based on these data, along with chemical-specific data describing physicochemical properties (Tables S2 and S3), *Ex Priori* calculates total release of each chemical ingredient to air, floor (dust), and skin. Then, using scenario/receptor specific data describing exposure scenario and human exposure factors (Table S4), *Ex Priori* models chemical exposure via inhalation, ingestion, and dermal routes. *Ex Priori* calculates the total absorbed dose across exposure routes, and applies a simple toxicokinetic model to predict the amount of chemical remaining in the body 24 h after exposure. A schematic diagram of *Ex Priori* is presented in Figure 1. Details of *Ex Priori*, including all model equations, are provided in the Supplemental Material. Variables calculated by *Ex Priori* are described in Table S5. A brief summary of *Ex Priori* is provided here.

Product composition data are collected from the Consumer Product Chemical Profiles database (CPCPdb) [22], a database of consumer product composition data derived from Material Safety Data Sheets made publicly available from a major retailer in 2015. CPCPdb contains information on 1797 chemicals found in 8921 consumer products. CPCPdb is part of the larger Consumer Product Database (CPDat); details on data collection, curation, and quality assurance for CPDat are described by Dionisio and colleagues [23]. *Ex Priori* uses 228 CPDat product categories, each describing a particular type of consumer product (for example, Antifreeze, Laundry Detergent, Body Wash, Insect Repellent, etc.). *Ex Priori* refers to these 228 CPDat product categories as “products”, envisioning each one as a single generic product (even though it likely includes data for multiple brands and/or varieties). For each product, *Ex Priori* uses CPDat product composition data consisting of the average mass fraction of chemical ingredients (grams of chemical per gram of product, averaged across brands/varieties within a product) [24]. In this way, *Ex Priori* models the average version of each generic product. If CPDat data did not indicate that any brands/varieties of a particular product contained a particular chemical, then the mass fraction of that chemical in that product is assumed to be zero. However, if *no* brands/varieties of *any* product contained a particular chemical—i.e., that chemical does not appear in CPDat as an ingredient for any of the consumer products included in this analysis—then that chemical was excluded from analysis altogether.

Consumer habits and practices data for each of the 228 CPDat “products” are harmonized with SHEDS-HT as presented in Isaacs et al. [6] By default, *Ex Priori* uses data reflecting the habits and practices of the average adult consumer. These habits and practices data include frequency of product use (events per year); mass of product used (grams per event); and duration of product use per event (minutes per event). These data are combined to calculate the total mass of each product used in grams per year and the total duration of product use in minutes per year. Then, these quantities are divided by 365 days per year, to yield the average mass of each product used per day (grams per day) and the average duration of product use per day (minutes per day), averaged over a full year.

Next, *Ex Priori* models chemical fate in the indoor environment: partitioning from the product(s) into the exposure media of air, skin, floor, and indoor dust, as shown in Figure 1. First, the mass of chemical that partitions into the air is calculated based on the mass of product used, the mass fraction of chemical in the product, the duration of product use, and a constant emissions rate estimated from the air-water partitioning coefficient. (See Supplemental Material S1.4.1 for details; *Ex Priori* is limited in scope to liquid formulations of consumer products, and is not applicable to solid articles.) Then, the remaining mass of chemical is assumed to partition between the floor and the skin, according to product-specific “floor factors” and “dermal factors”. The dermal factors are taken to be the percent of product retained on the skin after washing as used in SHEDS-HT [6]; the floor factors are generally taken as (100%—dermal factor). Then, the mass of chemical that partitions from the floor to indoor dust is calculated based on the assumed loading of indoor dust on the floor.

Then, *Ex Priori* models three key routes of human exposure: inhalation, dermal, and ingestion (see Figure 1).

Inhalation exposure is divided into three sub-routes: airborne direct exposure via inhalation of gases during product use, airborne indirect exposure via inhalation of gases after product use, and airborne indirect exposure via inhalation of dust particles after product use. See Supplemental Material (S1.4.1) for details.

Inhalation of gases (both during and after product use) is modeled using a two-zone model, which assumes a smaller volume of air constituting a near-field “user bubble” within a larger room (Figure S1) [25]. Air is exchanged between the user bubble and the larger room at flow rate β (m^3^/hour), and air is exchanged between the larger room and the outside with air exchange rate AER (changes/hour). Chemical is assumed to be emitted from a liquid product into the user bubble during product use, at a constant emission rate estimated using a steady-state assumption. An upper bound is placed on the constant emission rate such that the air concentration in the user bubble does not exceed the saturation concentration of the chemical in air. See Supplemental Material S3.3 for details. This approach to estimating emission rate was selected as a compromise between the highly conservative approach of assuming all chemical in the product is emitted into the air [26], and attempting to predict scenario- and product-specific time-dependent emission rates, which have high data and computational requirements that make them infeasible for rapid exposure modeling [27,28,29,30,31,32,33,34,35,36,37,38]. Inhalation of dust particles is modeled using the assumption that chemical mass that falls to the floor after use mixes with the dust on the floor and can then become resuspended and inhaled. Particle inhalation exposure is assumed to involve dust particles less than 2.5 microns in diameter (PM_2_._5_), which can penetrate deeply into the lung. Absorption of inhaled chemical is estimated using the blood:air partition coefficient estimated using the methodology of Buist, Wit-Bos [39].

Dermal exposure consists of dermal direct exposure via absorption of product residue on the skin, both during and after use. As mentioned above, product-specific “dermal factors” determine the fraction of product retained on the skin after use; these represent the fraction of product that remains on the skin after washing (if relevant), assuming that washing is an integral part of product use. That post-washing fraction of product is assumed to stay on the skin indefinitely after use; further wash-off is not modeled. See Supplemental Material (S1.4.2) for details. Dermal absorption is predicted as a function of molecular weight and octanol-water partitioning, according to the method of Weschler and Nazaroff [40].

Ingestion exposure consists of indirect ingestion only, divided into three sub-routes: ingestion indirect exposure via incidental ingestion of settled particles, ingestion indirect exposure via hand-to-mouth transfer of chemical on the skin, and ingestion indirect exposure via inhalation and subsequent ingestion of large resuspended particles deposited on the floor. (*Ex Priori* does not model dietary exposures, e.g., from food contact materials). See Supplemental Material (S1.4.3) for details. Oral absorption is predicted as a function of the octanol-water partition coefficient and the polar surface area according to the model of Linnankoski, Mäkelä [41].

Then, *Ex Priori* models toxicokinetics: how much of the absorbed dose is cleared from the body after one day, ultimately predicting the body burden that remains 24 h after exposure. See Supplemental Material (S1.5) for details. Half-lives are estimated as a function of the octanol-water partitioning coefficient, based on a previously published regression relation [42]. Finally, *Ex Priori* produces a list of chemicals ranked by the predicted body burden remaining 24 h after exposure; see Supplemental Material (S1.6) for details.

*Ex Priori* uses four different groups of inputs: (1) product-specific inputs including product composition data, habits and practices data, and dermal and floor factors; (2) chemical-specific inputs including physicochemical properties estimated from structure using the OPEn (Quantitative) Structure-activity/property Relationship App (OPERA) model [43] and toxicokinetic parameters measured or estimated using other quantitative structure-activity relationship (QSAR) models (see Supplemental Material S1.5 for details); (3) receptor inputs describing human exposure factors such as inhalation rate, skin surface area, and hand-to-mouth fraction; and (4) environmental inputs describing exposure factors such as building ventilation rate, dust load, and room size. Input parameters are detailed in Supplemental Material (S2), Tables S1–S4. The *Ex Priori* model spreadsheet is prepopulated with default inputs for 1108 chemicals, 228 consumer product categories, one default use scenario per product, one default human receptor, and one default indoor environment.

The 1108 chemicals with default chemical-specific inputs in *Ex Priori* consist of the subset of CPDat chemicals that could be mapped to a structure on the CompTox Chemicals Dashboard [44]. Some of these chemicals are mixtures or polymers; these were mapped to a single representative structure, and such cases are flagged in the model output (see Discussion). CPDat data indicating chemical occurrence in at least one product was required in order to estimate chemical release from products. A structure was required in order to allow QSAR predictions of physicochemical and toxicokinetic parameters. Chemicals that either did not occur in any products in CPDat, or had no available structure on the Comptox Chemicals Dashboard, were excluded from prioritization. Importantly, this list of 1108 chemicals is not intended to be an exhaustive list of all possible chemical exposures from consumer products. If a chemical does not appear on the list of 1108, its exposure potential is *not* assumed to be zero. Rather, these are the 1108 chemicals with sufficient data to allow *Ex Priori* to prioritize potential exposures.

Where possible, *Ex Priori* default inputs have been harmonized with existing EPA data and higher-tier exposure modeling efforts; sources for default input values are described in the Supplemental Material, Tables S1–S4. Where scenario-specific data are known or required, the user can overwrite any default input variables by editing the corresponding cell in the Excel spreadsheet.

### 2.1. Product-Category Weights

By default, *Ex Priori* models the baseline exposure scenario of an average adult consumer, including average consumer habits and practices. However, consumer habits and practices vary—often in ways that affect exposure for entire product categories. Changes in consumer habits and practices may give rise to larger public health questions about chemical exposures. For example, during the COVID-19 pandemic, consumers are spending more time inside the home [45]. They have reduced their use of personal care products: in 2020 vs. 2019, Americans spent 18% less money on personal care products [46], and spent 10% less time on grooming [45]. However, consumers have increased their use of cleaning and home maintenance products: in 2020 vs. 2019, Americans spent 9% more money on “housekeeping supplies” [46], spent 30% more time on lawn and garden care, and spent 12% more time on housekeeping [45]. These changes in consumer habits and practices raise a public health question: how do chemical exposures change in this pandemic scenario of habits and practices?

In order to rapidly model changes in habits and practices, *Ex Priori* groups the 228 CPDat products into nine product categories: Arts and Crafts, Auto Products, Home Maintenance, Home Office, Inside the Home, Landscape/Yard, Personal Care, Pesticides, and Pet Care. (For example, the “Personal Care” product category includes CPDat products such as Body Wash, Bar Soap, Shaving Cream, Hairspray, Deodorant, etc.) For each of these product categories, *Ex Priori* assigns a product-category weight, which scales the default (average) daily use for all products within a category with one click. Conceptually, this weight can represent a change in amount, frequency, and/or duration of use for all products in a category. The product-category weights conceptually assume that consumers uniformly increase or decrease product use for all products in a category. The weight values typically reflect order-of-magnitude level changes in product use, in keeping with the prioritization-level nature of *Ex Priori*. For example, a weight of 0.1 reflects low-normal use of products in a category; a weight of 10 represents high-normal use; a weight of 100 represents very heavy use, e.g., that of a hobbyist.

To model the reduced use of personal care products in the COVID-19 pandemic situation described above, the “Personal Care” product category weight was set to 0.5, reflecting an assumption that consumer use of personal-care products has been cut in half. To model the increased use of cleaning and DIY products and the increased time spent inside the home, the “Home Maintenance,” “Inside the Home,” and “Landscape/Yard” product category weights were all set to 10, reflecting an assumption that consumer use of these product categories has increased by an order of magnitude. These product category weights are intended only as an illustrative order-of-magnitude approximation of the changes in consumer use suggested by the data cited above [45,46]. To return to the baseline scenario of the average adult consumer, all product-category weights can be reset to 1.

With each change to a product-category weight, *Ex Priori* will instantly recalculate potential exposures and produce a new chemical ranking for the new exposure scenario. By adjusting *Ex Priori* controls for different scenarios, all 1108 consumer product ingredients are simultaneously reprioritized on anticipated body-burden, enabling a fast representation of day-to-day personal multi-chemical exposures from consumer products for varying exposure scenarios.

To illustrate the power of product category weights to rapidly answer a public health question about chemical exposure, *Ex Priori* was run twice: once with the default scenario of the average adult consumer (all weights set to 1), and once for a pandemic scenario (with the Personal Care product-category weight set to 0.5 and “Inside the Home”, “Home Maintenance”, and “Landscape/Yard” product-category weights set to 10). Changes in chemical prioritization were observed in the two different scenarios.

### 2.2. Pathway Weighting

To facilitate user exploration of the importance of different routes of exposure, *Ex Priori* also includes “pathway switches.” Currently, these weights are implemented as checkboxes that allow the user to turn each main exposure route (dermal, ingestion, and inhalation) “on” or “off.” The “pathway switches” allow the user to explore how chemical prioritization changes if, e.g., dermal exposures are disregarded, or inhalation exposures are disregarded.

### 2.3. Sensitivity Analysis

A one-way discrete sensitivity analysis was conducted for selected *Ex Priori* input parameters to evaluate the impacts of these parameters on absorbed dose (not remaining body burden) by exposure routes (dermal, ingestion, and inhalation). Eight candidate variables were selected for the analysis because they define the indoor environment or individual exposure factors, and therefore may vary substantially between individual people and homes. The selected parameters, their default values, and their ranges are listed in Table 1. For each model iteration, only one parameter is perturbed by replacing its default value with an alternative (either low or high, as defined in Table 1), while other values of other parameters are set to the defaults. To perform the sensitivity analysis directly within the *Ex Priori* spreadsheet tool, Microsoft Excel’s “What-If Analysis” functionality was used (Microsoft Excel for Microsoft 365, version 16.0; © Microsoft Corporation; Redmond, WA, USA). For each model iteration, the following summary statistics were calculated for the log_10_-scaled absorbed amounts for each exposure route, and for the log_10_-scaled total amount absorbed: median; 25th and 75th percentiles (denoted Q1 and Q3, respectively); the lowest value greater than or equal to Q1 − 1.5 × (Q3–Q1); and the highest value less than or equal to Q3 + 1.5 × (Q3–Q1). (The last two statistics are the usual “whiskers” on a standard box-and-whisker plot.) Model sensitivity to each parameter was evaluated qualitatively, by examining shifts in the distribution summarized by these statistics.

### 2.4. Evaluation Using Exposures Inferred from NHANES Biomonitoring Data

The predictions of *Ex Priori* were evaluated by comparing them to median population aggregate exposures inferred for parent chemicals of biomarkers measured in urine samples from the U.S. population by the National Health and Nutrition Examination Survey (NHANES) [47]. There were 42 chemicals (identified by CASRN) that had both *Ex Priori* exposure predictions and NHANES-inferred exposures; the comparison was made for these 42 chemicals. Specifically, *Ex Priori* was run with all product category and pathway weights set equal to 1. The *Ex Priori*-predicted amount absorbed (not amount remaining after 24 h) was converted from mass units of g/day to dose units of mg/kg/day (by assuming an average adult body weight of 70 kg and a conversion factor of 1000 mg/g). The resulting *Ex Priori*-predicted absorbed doses were compared to the median NHANES-inferred exposures specifically for the age 20–65 demographic group. The *Ex Priori* amount absorbed was selected for comparison, rather than the *Ex Priori* amount remaining after 24 h, because amount absorbed was the quantity conceptually most similar to the quantity inferred by Stanfield and colleagues [47]: they inferred aggregate exposures to the parent chemicals of NHANES analytes, in mg/kg/day, which they interpret as “equivalent oral dose assuming 100% oral absorption.” Stanfield and colleagues [47] inferred exposures for several different demographic groups; we chose to compare to NHANES-inferred aggregate exposures for adults ages 20–65, since *Ex Priori* is intended to represent an adult consumer. To quantify the comparison, ordinary least-squares linear regression was used: log10-transformed NHANES-inferred aggregate exposures were regressed on log10-transformed *Ex Priori*-predicted absorbed doses. Analysis was conducted using R (version 4.2.0, R Foundation for Statistical Computing, Vienna, Austria).

## 3. Results

### 3.1. Chemical Rankings

**Figure 2:**
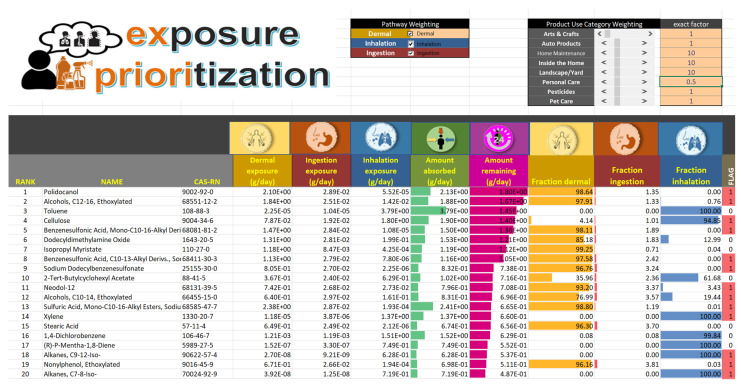
Screenshot of a portion of the “Dashboard” tab of the *Ex Priori* model spreadsheet, showing an illustrative example of chemical rankings and other model outputs.

*Ex Priori’s* ultimate output is chemical rankings based on the model-predicted body burden remaining after 24 h. The results can be used to test the impact of various user inputs on the final rankings. An example screenshot of the output on the “Dashboard” tab of the *Ex Priori* spreadsheet is shown in Figure 2. *Ex Priori* shows not only the chemical rankings, but also the model-estimated absorbed dose by each route (dermal, inhalation, and ingestion), the model-estimated total absorbed dose, and the model-estimated amount remaining in the body after one day. As well, *Ex Priori* shows the fraction of absorbed dose attributable to each route, visualized with a data bar formatting for at-a-glance examination of relative route importance. Finally, *Ex Priori* shows flags indicating whether each chemical is a mixture or a polymer, in which case results may be more uncertain (see Discussion).

Example results are shown in Table 2 (the baseline scenario) and Table 3 (the pandemic scenario). To assess the effect of including toxicokinetic considerations, Table 2 and Table 3 also present what the chemical rankings would have been if they were based on absorbed dose only (without toxicokinetic considerations), rather than on remaining body burden after 24 h (with toxicokinetic considerations).

For the default (average) exposure scenario (Table 2), the highest-ranking chemicals have exposures primarily driven by the dermal route. Most of their mass occurs in the Personal Care product category, where dermal factors tend to be high, which makes the skin loading relatively higher. They tend to be lipophilic (high log K_ow_) and not highly volatile (low log Henry’s law coefficient), which makes them relatively more permeable through skin. Additionally, their half-lives tend to be longer than 24 h, so that most of the absorbed dose remains in the body after 24 h. Comparing the rankings by remaining body burden vs. absorbed dose shows the importance of considering toxicokinetics in chemical prioritization. Most of these chemicals would not rank in the top 20 if only absorbed daily intake were considered—but when their longer half-lives are considered, these exposures ultimately result in higher body burdens.

Table 3 shows the new top 20 ranked chemicals under the pandemic scenario. For comparison, Table 3 also shows the rankings of these chemicals under the baseline scenario. In the pandemic scenario, chemicals found primarily in Inside the Home products rise 20, 30, or more places in the rankings compared to baseline, reflecting that chemicals from this product category become more prominent in overall exposure when use of Personal Care products is reduced. Moreover, in contrast with the nearly 100% dermal exposures for the top 20 chemicals in the baseline exposure scenario, the inhalation route starts to become important for some chemicals in the pandemic scenario. For Toluene, nearly 100% of exposure occurs via inhalation; for 2-Tert-Butylcyclohexyl Acetate (found in Inside the Home air fresheners and disinfectants), approximately 62% of exposure occurs via inhalation.

These results show that, with the product category weights, a single click can model a change in individual use patterns—e.g., a pandemic scenario with more time spent at home and less personal grooming—and instantly show the resulting changes in the chemicals that are prominent in overall exposure, including chemicals from different product sources and different routes of exposure.

### 3.2. Sensitivity Analysis

**Figure 3:**
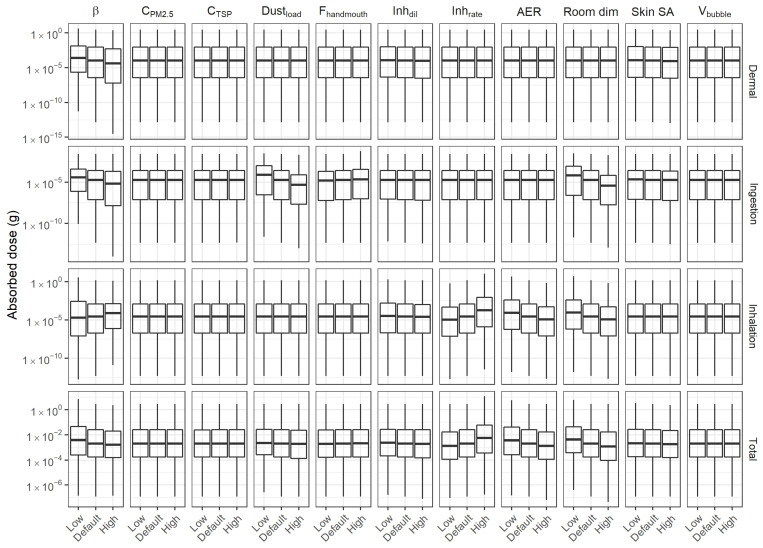
Outputs of one-way sensitivity analysis. Each row represents one exposure route (the bottom row represents total exposure). Each column represents one model parameter. β: Air flow rate between user bubble and larger room (m^3^/hour). C_PM2.5_: Background indoor PM2.5 concentration (µg/m^3^). C_TSP_: Background indoor total suspended particulate concentration (µg/m^3^). Dust_load_ = Mass of dust on the floor per unit area (g/m^2^). F_handmouth_ = Fraction of chemical transferred from hand to mouth (unitless). Inh_dil_ = Dilution factor for products used outdoors (unitless). Inh_rate_ = Inhalation rate (m^3^/day). AER = Building air exchange rate (residential) (# air changes/hour). Room dim = Dimension of one side of square room (m). Skin SA = Skin surface area (m^2^). V_bubble_ = Air volume of near-field user bubble (m^3^). Within each panel, three box-and-whisker plots represent the distribution of intakes (absorbed doses) via the specified route at the low, default, and high values of the specified parameter (as marked on the horizontal axis). Low, default, and high values for each parameter are defined in Table 1. Lower and upper hinges correspond to the first and third quartiles (the 25th and 75th percentiles). The upper whisker extends from the hinge to the largest value no further than 1.5 × IQR (distance between the first and third quartiles) from the hinge. The lower whisker extends from the hinge to the smallest value at most 1.5 × IQR of the hinge.

Results of the discrete one-way sensitivity analysis are visualized in Figure 3, which has three rows, representing dermal, ingestion, and inhalation routes, and eight columns, representing the eight variables assessed in the analysis. The horizontal axis for each panel is labeled “low”, “default,” and “high”, indicating which value for the variable was used (low, default, and high values for each variable are defined in Table 1). The vertical axis presents log_10_ transformed absorbed doses in units of grams. Note that this sensitivity analysis is performed using absorbed dose, not ADME output (amount remaining in body after one day), because *Ex Priori* does not currently apportion ADME outputs among routes. Within each panel, the boxplots for “low” and “high” values can be compared to the boxplot for the “default” value. If the position is shifted up or down compared to the “default” boxplot, then the perturbed parameter impacts the absorbed dose estimated for that route.

For dermal exposure (top row in Figure 3), most of the parameters evaluated do not impact absorbed dose, with the exception of the air flow between user bubble and room (denoted β). The sensitivity to β occurs because the fraction of chemical apportioned to the air depends on β, and only the remaining fraction of chemical *not* apportioned to the air is available for dermal absorption (see Supplemental Material S1.4.2 and S3.4). A larger β means that more chemical mass is apportioned to the air, and proportionally less chemical mass is available to the skin. Interestingly, dermal exposure is not sensitive at all to skin surface area. This result occurs because, for the majority of chemicals included in *Ex Priori* (809 out of 1108), the predicted total flux through the skin exceeds the upper limit of the total mass of chemical present on the skin; for these chemicals, the absorbed amount is simply assumed to be the total mass of chemical present on the skin, and is not affected by skin surface area at all. Predicted dermal exposures often exceed the upper limit because *Ex Priori* currently does not model any pathway for removing chemicals from skin. This is also the reason why dermal exposures dominate other routes for most chemicals.

For ingestion exposure (second row in Figure 3), absorbed dose is reduced under four conditions: (1) a higher air flow between user bubble and room (β); (2) a larger dust load, (3) a smaller hand-to-mouth fraction, and 4) a larger room dimension (length and width). Ingestion exposure is driven by incidental dust ingestion; therefore, exposure is reduced by conditions that contribute to more dilution of the chemical in room dust (conditions 1, 2, and 4) and lesser dust ingestion (condition 3).

For inhalation exposure (third row in Figure 3), the results indicate that absorbed dose is increased by higher β and higher inhalation rate, and decreased by higher room air exchange rate (AER) and room dimensions. The increase with inhalation rate is intuitive: breathing more air produces greater inhalation exposure. The increase with β is somewhat counterintuitive, since increased β means that air in the user bubble is exchanged (and thus chemical is removed from the air) faster. However, as previously discussed, the fraction of chemical apportioned to the air increases with β; on balance, for the median chemical in *Ex Priori*, this increase outweighs the increased rate of chemical removal from the user bubble. This result is explained by the fact that, at median β, the fraction of total chemical emitted to the air is small (less than 0.1) for the majority of chemicals in *Ex Priori*. As shown in the Supplemental Material (Section S3.4, Table S6, Figures S2–S6), when the fraction of chemical emitted is near 1 at median β (which typically occurs for volatile chemicals), then further increasing β does not allow any additional chemical to be emitted to the air (because all of it is already in the air). In this case, the increased removal of chemical from the air at increased β ultimately results in decreased inhalation exposure. However, when the fraction of chemical emitted is less than about 0.7 at median β, then increasing β above the median effectively allows more of the chemical to be emitted to the air (see the Supplemental Material, Figure S7). This effect outweighs the increased removal of chemical from the air with increased β, ultimately resulting in increased inhalation exposure. The effect is even stronger when the chemical reaches its air saturation concentration (thus reaching a cap on the amount that can be emitted) at median β; increasing β will lift the saturation-imposed cap on emission. The majority of chemicals in *Ex Priori* have fraction emitted less than 0.1 at median β (see the Supplemental Material, Figure S7), and 56% of chemicals reach air saturation concentration at median β. Therefore, for the median chemical in *Ex Priori*, an increase in β results in increased inhalation exposure. By contrast, increasing the AER for the larger room has the expected effect of decreasing inhalation exposure. Increasing the room dimensions has the effect of diluting the same chemical mass in a larger volume of air, and has little or no effect on the fraction of chemical that can be emitted to air, thus reducing inhalation exposure.

The bottom row shows all three routes combined, to assess sensitivity of the total absorbed exposure. Total exposure is most sensitive to β, inhalation rate, room AER, and room dimension. This result reflects the nature of *Ex Priori*’s exposure models: chemical partitioning to air is considered first, and only the remaining chemical mass after air partitioning is available for dermal or ingestion exposure.

This qualitative, one-way, discrete sensitivity analysis of a subset of parameters does not rule out possible sensitivity to other parameters, nor to combinations of parameters. It does not analyze sensitivity to chemical- or product-specific inputs, and it does not characterize distributions of model predictions corresponding to data-driven distributions of model parameters. However, it does reveal key features of *Ex Priori*’s exposure models that are important for context when interpreting prioritization results: namely, factors affecting chemical partitioning to air will change not only inhalation exposures, but also dermal and ingestion exposures.

### 3.3. Evaluation by Comparing to NHANES-Inferred Exposures

**Figure 4:**
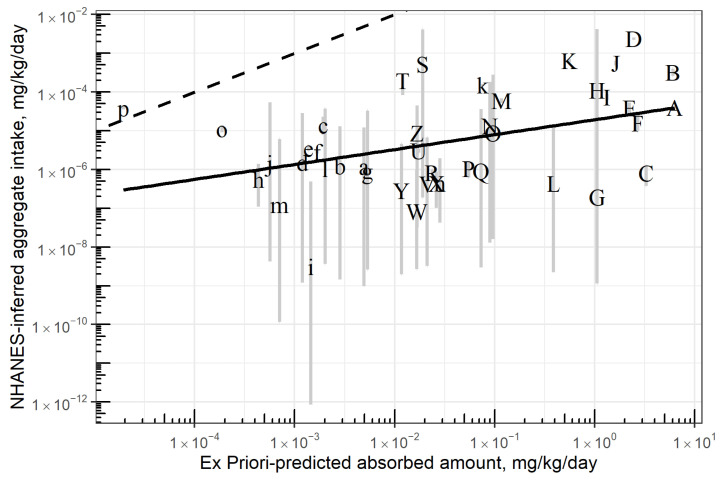
NHANES-inferred aggregate exposure vs. *Ex Priori*-predicted absorbed amount, each in mg/kg/day, on log10-log10 scale, for 42 chemicals. Each letter represents one chemical; lower-case letters represent different chemicals from their corresponding upper-case letters (e.g., “A” and “a” represent two different, unrelated chemicals). Gray vertical line segments represent range of 95% credible interval bounds on NHANES-inferred median aggregate exposures. Dashed diagonal line represents the identity line, y = x. Solid diagonal line represents the best-fit linear regression model, i.e., log10(y) = −4.7 + 0.38 log10(x), where y = NHANES-inferred aggregate exposure and x = *Ex Priori*-predicted absorbed amount converted to mg/kg/day. See text for details on linear regression. See Supplemental Material Table S7 to map letter codes to chemical names and CASRN.

The comparison between *Ex Priori*-predicted absorbed amount and NHANES-inferred aggregate exposures [47] is visualized in Figure 4. The best-fit linear model was log10(y) = −4.7 + 0.38 log10(x), where y = NHANES-inferred aggregate exposure and x = *Ex Priori*-predicted absorbed amount. Adjusted R^2^ for this linear model was 0.15. See Supplemental Material Section S4.

This result implies that *Ex Priori* estimates of absorbed dose are biased high—not unexpected, given the known-conservative assumptions of *Ex Priori*. However, given that the intended use of *Ex Priori* is screening-level prioritization, the more important question is whether *Ex Priori’s* prioritization correlates with the prioritization implied by the NHANES inferences. One metric answering this question is the R^2^ of the comparison, which represents correlation between *Ex Priori* predicted exposures and NHANES-inferred exposures. A higher R^2^ represents greater correlation and therefore more-similar prioritization. The R^2^ of the comparison was 0.15. To contextualize this result, we compared it to the R^2^ of a comparison made by Stanfield and colleagues [47], between the NHANES-inferred exposures and the predictions of another rapid prioritization-level exposure model, SEEM3. We used information reported in Supplemental Table S9 of [47], which reports NHANES-inferred exposures and SEEM3-predicted exposures for 37 chemicals. (SEEM3 [57] is a machine-learning model trained on exposures inferred from NHANES biomonitoring data available in 2014. The 37 chemicals reported by Stanfield and colleagues [47] are chemicals that are newly added to NHANES urine biomonitoring since the development of SEEM3, and were therefore not in the SEEM3 training set.). Specifically, we subset that table to retain only the NHANES-inferred exposure for the most-recent available NHANES cohort for each CASRN, then performed an unweighted linear regression of log10(NHANES-inferred exposure) vs. log10(SEEM3-predicted aggregate intake). The resulting best-fit model was log10 (NHANES) = 0.75 + 1.06 log10 (SEEM3), with adjusted R^2^ = 0.15. See Supplemental Material section S4. (We performed an unweighted regression, unlike the weighted regression reported by Stanfield and colleagues [47], in order to better compare with the unweighted regression of NHANES-inferred exposures vs. *Ex Priori* predictions.). In other words, *Ex Priori* predictions correlate with NHANES-inferred exposures about as well as SEEM3 predictions do.

## 4. Discussion

Evaluating multi-chemical exposure modelssuch as *Ex Priori* remains a challenge due to the complexity of human behaviors and chemical fate and transport. The fate resulting after product use results from chemical partitioning and transformation that occur both in the indoor environment and within the body [17]. This complexity is acknowledged as a limitation in exposure science [58].

Sensitivity analysis indicates that the model is sensitive to parameters that determine the fraction of chemical mass that partitions into the air, because that affects the chemical mass available to other routes of exposure. The chemical mass partitioning to air is governed by the estimated constant emission rate during product use. Limitations of the assumption of constant emissions rate, and of the approach used to derive the estimated emission rate, therefore strongly affect the predictions of *Ex Priori*. These assumptions also affect the sensitivity to other model parameters, particularly β. A more-detailed time-dependent emissions model would likely be more realistic; however, a time-dependent emissions model would suffer from data limitations (as it would require detailed information about how each product is applied and used, which is unavailable for most products), and would present problems with computational tractability (numerical solution of systems of differential equations for thousands of chemicals is impractical in an Excel-based modeling tool). The constant emissions rate is an imperfect approximation, but it represents a compromise between the extremely conservative typical default assumption that *all* chemical in the product is emitted to the air, and the currently infeasible approach of modeling detailed time-dependent air emissions. Additionally, *Ex Priori* models emissions with the assumption that products are liquid, meaning that it is not applicable to solid consumer products or articles. Improving estimation of the emissions rate is a key area for future refinement of the model (A much more detailed discussion of chemical emissions rate assumptions and uncertainties is included in the Supplemental Material, Sections S3.3 and S3.4).

Similarly, sensitivity analysis reveals that dermal exposure is not sensitive at all to skin surface area. This uncertainty represents a limitation of the model’s simplified representation of dermal uptake, another key area for future model refinement. Moreover, *Ex Priori* currently uses a simplified model of product wash-off by assuming that a constant fraction of product remains on the skin after use, where that fraction represents the fraction retained post-wash-off for each product type, as derived in SHEDS-HT. Time-dependent wash-off is not modeled. This simplification likely results in conservative dermal exposure estimates.

Another source of uncertainty stems from *Ex Priori’s* reliance on physicochemical parameters based on chemical structure. This presents challenges when a representative chemical structure is difficult to identify, such as for chemical mixtures or for polymers. For example, “Alcohols, C12-16, Ethoxylated” (CAS-RN 68551-12-2) represents a mixture of fatty alcohols with different structures; assigning a single representative structure and single set of representative physicochemical properties is therefore necessarily uncertain. For another example, cellulose (CAS-RN 9004-34-6) is a polysaccharide, and the chemical structure used to find the representative physicochemical properties represents only one unit of the polysaccharide chain—thus volatility is overestimated, leading to the probably unrealistically high inhalation exposure predicted in Table 3. In future work, it might be possible to approach quantifying the uncertainty for polymers and mixtures by enumerating possible structures for these substances and performing sensitivity analysis for the possible structures. Improved curation of representative chemical structures and of physicochemical properties is a key area for future refinement of *Ex Priori*. In the meantime, *Ex Priori* flags mixtures and polymers in its output (see Figure 2), allowing users to quickly identify these substances and decide whether and how to include them in any decision-making that uses *Ex Priori* output. All input parameters are user-editable, so if a user identifies better representative physicochemical properties for a flagged chemical, it is easy to enter them into the model spreadsheet and re-run *Ex Priori* using the new values.

Another limitation of *Ex Priori* is its default product composition database. CPCPdb is based on data gathered in 2015, and therefore may not reflect the most recent products and ingredients available in the fast-moving consumer products landscape. For this analysis, product composition data were not evaluated against external sources. Data curation efforts for CPDat (including product composition data) are ongoing. However, the flexible, modular, user-accessible nature of *Ex Priori* allows the user to add or substitute product composition data as desired, if the user has a preferred source for such data.

The sensitivity analysis presented here does not explore sensitivity to product compositional data (mass fractions of chemicals in products). This is acknowledged as a limitation; however, a complete sensitivity analysis, including high, medium, and low-end mass fractions for each chemical in each product, is beyond the scope of the current work. However, such a sensitivity analysis is of high interest for future work. In general, *Ex Priori*-predicted exposures to a given chemical will scale linearly with total mass of chemical across products, until the chemical mass is great enough that chemical emissions to air inside the “user bubble” cause the air concentration to reach its saturation limit such that no additional chemical can partition into the air. Then, inhalation exposures will cease to scale up with total chemical mass. However, dermal and ingestion exposures will scale up more quickly, as a smaller fraction of chemical will be lost to the air, and therefore a greater fraction of chemical will remain available to partition onto the skin and onto floor dust. For quantitative analysis of the effect of variability in product composition, along with variability in consumer use patterns and other exposure factors, we recommend the more-detailed population exposure model SHEDS-HT [6].

Despite these acknowledged limitations, *Ex Priori* appears to perform comparably to another rapid exposure model (SEEM3) when the predictions of both models are evaluated by examining their correlation to median aggregate exposure rates for the U.S. population inferred from NHANES urine biomonitoring data [47].

## 5. Conclusions

*Ex Priori* shows promise as a screening-level chemical prioritization tool designed to allow exposure modelers to rapidly explore various patterns of consumer behavior and their potential impacts on exposure. *Ex Priori* can be used as an exploratory scoping tool before using more-detailed EPA exposure models and tools such as SHEDS-HT [6] and/or more-comprehensive life cycle exposure models such as RAIDAR [20] and USETox [15]. *Ex Priori* considers multi-chemical exposures from consumer products and articles accounting for product formulation and use; physical-chemical properties, such as partition coefficients; and user exposure factors and activity patterns. The power of *Ex Priori*’s simple dashboard is the ease of dynamically exploring exposure scenarios, variables, and routes and their ramifications on multi-chemical exposures.

## Figures and Tables

**Table 1:** Parameters selected to conduct one-way sensitivity analysis.

Parameter	Definition	Range			Source of Default Values	Source of Low/High Values
		Low	Default	High		
β	Air flow rate between user bubble and larger room (residential)	60 m^3^/h	82.008 m^3^/h	300 m^3^/h	United States Environmental Protection Agency [48]	Derived from Zhang, Banerjee [49] (see Supplemental Material S3.4; see also [27,49,50]
CPM2.5	Background indoor PM2.5 concentration	5 µg/m^3^	7.16 µg/m^3^	9 µg/m^3^	Deshpande, Frey [51]	Deshpande, Frey [51]
CTSP	Background indoor PM10 concentration	40 µg/m3	75 µg/m^3^	150 µg/m^3^	Assumed (half of NAAQS standard for PM10 [52], as a rough estimate)	Assumed (vary default by a factor of 2 in either direction)
Dust_floor_load	Mass of dust on the floor/unit area	0.1 g/m^2^	0.52 g/m^2^	2.5 g/m^2^	Wilson, Jones-Otazo [53]	Wilson, Jones-Otazo [53]
Frachand_mouth	Fraction of chemical that is transferred from hand to mouth	0.05	0.2	0.8	Ozkaynak, Xue [54]	Ozkaynak, Xue [54]
Inhdil	Dilution factor to account for increased ventilation and decreased exposure when using a product outdoors	1	20	100	Estimated based on Klepeis, Gabel [55]	Estimated based on Klepeis, Gabel [55]
Inhrate	Volumetric breathing rate	6.8 m^3^/day	16.2 m^3^/day	71.2 m^3^/day	EPA [56]	EPA [56] (low value is average of age groups ≥ 21 for sedentary/resting; high value is average of age groups ≥ 21 for high intensity)
AER	Building air exchange rate (residential)	0.1 air changes/h	0.45 air changes/h	3 air changes/h	EPA [56]	EPA [56]
Room dimension	Dimension of one side of square room	2.8 m	5.8 m	14.2 m	EPA [56]	EPA [56]
Skin SA	Skin surface area of adult human	1.61 m^2^	1.95 m^2^	2.425 m^2^	EPA [56]	EPA [56] (low value is average of 5th percentile for adults; high value is average of 95th percentile for adults)
Vbubble	Near field volume during product use (user “bubble” as compared to room volume)	0.125 m^3^	0.2 m^3^	27 m^3^	Nicas [25]	Assumed

**Table 2:** Subset of chemical rankings (top 20 of 1108), with rankings by exposure route. All route and product category weights are set to 1, and all parameters take their default value. ((*) = polymers and/or mixtures; does not necessarily apply to each component of a mixture and does not apply to a single sub-unit of a polymer).

		*Ex Priori* Rank (out of 1108 Chemicals) Based on…		Percent of Absorbed Dose via Route					
Chemical Name	CASRN	Body Burden after 24 h	Absorbed Daily Intake	Dermal	Ingestion	Inhalation	Log10 Kow	Log10 Henry’s Law	Half-Life (Hours)
Alcohols, C12-16, Ethoxylated *	68551-12-2	1	1	99.14	0.35	0.51	5.90	−4.45	141.94
Isopropyl Myristate	110-27-0	2	4	99.93	0.06	0.02	6.90	−6.12	274.90
2-Octyldodecan-1-Ol	5333-42-6	3	20	99.85	0.15	<0.01	8.83	−6.33	987.02
Decanoic Acid, Ester With 1, 2, 3-Propanetriol Octanoate *	65381-09-1	4	15	99.80	0.20	<0.01	4.97	−7.39	76.27
2-Ethylhexyl Salicylate	118-60-5	5	13	>99.99	<0.01	<0.01	4.05	−6.92	41.60
2-Cyano-3,3-Diphenyl-2-Propenoic Acid, 2-Ethylhexyl Ester	6197-30-4	6	22	99.97	0.03	<0.01	5.25	−6.62	91.77
Isopropyl Palmitate	142-91-6	7	26	99.98	<0.01	0.02	8.07	−6.60	598.89
Polyethylene Glycol Monostearate *	9004-99-3	8	27	>99.99	<0.01	<0.01	7.60	−7.23	438.16
Cetyl Alcohol	36653-82-4	9	25	99.91	0.08	0.01	6.59	−5.18	223.78
Tetradecan-1-Ol, Propoxylated, Esters With Propionic Acid *	74775-06-7	10	30	>99.99	<0.01	<0.01	7.61	−6.63	439.35
Stearic Acid	57-11-4	11	32	98.09	1.91	<0.01	8.08	−7.66	600.94
2-Ethylhexyl Palmitate	29806-73-3	12	34	97.25	2.75	<0.01	9.47	−6.64	1514.11
Stearic Acid, Monoester With Glycerol *	31566-31-1	13	31	99.88	0.12	<0.01	6.11	−7.53	163.14
Masoprocol	500-38-9	14	19	>99.99	<0.01	<0.01	3.55	−5.78	29.85
Cetostearyl Alcohol *	67762-27-0	15	41	99.89	0.10	0.01	7.88	−5.21	525.20
Celgard *	9003-07-0	16	45	98.13	1.87	<0.01	8.75	−7.13	934.75
4-Tert-Butyl-4′-Methoxydibenzoylmethane	70356-09-1	17	35	>99.99	<0.01	<0.01	4.64	−3.87	61.43
Alcohols, C16-18, Ethoxylated *	68439-49-6	18	46	97.60	2.35	0.05	9.09	−6.27	1171.97
Homosalate	118-56-9	19	28	>99.99	<0.01	<0.01	3.92	−6.92	38.08
Pramocaine Hydrochloride	637-58-1	20	36	99.99	<0.01	0.01	4.04	−7.58	41.38

**Table 3:** For the pandemic scenario: Subset of chemical rankings (top 20 of 1108), with rankings by exposure route. All other weights (route and product category) are set to 1, and all parameters take their default values. ((*) = polymers and/or mixtures; does not necessarily apply to each component of a mixture and does not apply to a single sub-unit of a polymer).

		*Ex Priori* Rank (out of 1108 Chemicals) Based on…		Rank in Baseline Scenario Based on…	Percent of Absorbed Dose via Route…					
Chemical Name	CASRN	Body Burden after 24 h	Absorbed Daily Intake	Body Burden after 24 h	Dermal	Ingestion	Inhalation	Log10 Kow	Log10 Henry’s Law	Half-Life (Hours)
Polidocanol *	9002-92-0	1	12	30	98.64	1.35	<0.01	5.36	−4.85	98.75
Alcohols, C12-16, Ethoxylated *	68551-12-2	2	16	1	97.91	1.33	0.76	5.90	−4.45	141.94
Toluene	108-88-3	3	4	44	<0.01	<0.01	>99.99	2.73	−2.23	17.33
Cellulose *	9004-34-6	4	15	38	4.14	1.01	94.85	4.46	−2.10	54.64
Benzenesulfonic Acid, Mono-C10-16-Alkyl Derivs., Sodium Salts *	68081-81-2	5	23	48	98.11	1.89	<0.01	6.15	−6.61	167.32
Dodecyldimethylamine Oxide	1643-20-5	6	20	32	85.18	1.83	12.99	4.86	−4.33	71.28
Isopropyl Myristate	110-27-0	7	32	2	99.25	0.71	0.04	6.90	−6.12	274.90
Benzenesulfonic Acid, C10-13-Alkyl Derivs., Sodium Salts *	68411-30-3	8	33	57	97.58	2.42	<0.01	6.15	−6.61	167.32
Sodium Dodecylbenzenesulfonate *	25155-30-0	9	42	35	96.76	3.24	<0.01	5.88	−6.57	140.00
2-Tert-Butylcyclohexyl Acetate	88-41-5	10	36	41	35.96	2.36	61.68	4.24	−3.64	47.04
Neodol-12 *	68131-39-5	11	46	65	93.20	3.37	3.43	5.90	−4.45	141.94
Alcohols, C10-14, Ethoxylated *	66455-15-0	12	43	36	76.99	3.57	19.44	5.29	−3.39	94.26
Sulfuric Acid, Mono-C10-16-Alkyl Esters, Sodium Salts *	68585-47-7	13	9	54	98.80	1.19	0.01	2.29	−6.51	12.93
Xylene *	1330-20-7	14	27	72	<0.01	<0.01	>99.99	3.14	−2.17	22.77
Stearic Acid	57-11-4	15	53	11	96.30	3.70	<0.01	8.08	−7.66	600.94
1,4-Dichlorobenzene	106-46-7	16	22	83	0.08	0.08	99.84	2.86	−4.93	18.91
(R)-P-Mentha-1,8-Diene	5989-27-5	17	49	66	<0.01	<0.01	>99.99	4.46	−1.50	54.55
Alkanes, C9-12-Iso-*	90622-57-4	18	58	91	<0.01	<0.01	>99.99	5.47	−0.83	106.30
Nonylphenol, Ethoxylated *	9016-45-9	19	51	55	96.16	3.81	0.03	4.43	−5.24	53.29
Alkanes, C7-8-Iso- *	70024-92-9	20	50	95	<0.01	<0.01	>99.99	4.09	−0.39	42.74

## Data Availability

Data are contained within the Supplementary Material.

## Supplementary Materials

The following supporting information can be downloaded at: https://www.mdpi.com/article/10.3390/toxics10100569/s1, *Ex Priori* Supplemental Material.docx (including the following: a detailed description of model including all equations; tables of *Ex Priori* inputs and calculated variables; detailed derivation of emissions assumption used in two-zone inhalation model; sensitivity analysis of inhalation exposure to β). Ex_priori_workbook.xlsb (Excel workbook implementation of *Ex Priori* model, including all data). Reference [59] is cited in the Supplemental Materials.
