# Supplementary material for: Exposure Prioritization (Ex Priori): A Screening-Level High-Throughput Chemical Prioritization Tool
Source: Toxics. 2022 Sep 28;10(10):569. doi: 10.3390/toxics10100569 (PMC9609548; doi:10.3390/toxics10100569)
Supplement: Supplementary file 1 [file toxics-10-00569-s001.zip › Ex Priori Supplemental Material.pdf]

Exposure Prioritization (Ex Priori): A Screening Level High throughput Chemical Prioritization Tool

S1. Ex Priori Model Structure
S1.1. Product-specific Inputs
S1.1.1. Product Composition
S1.1.2. Habits and Practices: Mass, Frequency, and Duration of Product Use
S1.1.3. Dermal and Floor Factors and Outdoor Dilution Factors
S1.2. Chemical-specific Inputs
S1.3. Receptor and Environmental Inputs
S1.4. Exposure Pathways
S1.4.1. Inhalation
S1.4.2. Dermal Direct
S1.4.3. Ingestion
S1.5. Estimation of Internal Dose
S1.6. Chemical Rankings
S2. Tables of Ex Priori Model Inputs and Calculated Variables
S3. Two-zone Model
S3.1. Inhalation Exposure during Use
S3.1.1. Cumulative Inhalation Exposure during Product Use
S3.2. Inhalation Exposure after Product Use
S3.2.1. Cumulative Inhalation Exposure after Product Use
S3.3. Estimating Emissions Rate
S3.3.1. Conservative Approach: Assume all Chemical is Emitted
S3.3.2. Most detailed approach: Time-dependent Emissions Models
S3.3.3. Compromise approach: Steady-state Scenario
S3.4. Sensitivity of Inhalation Exposure to β
S4. Evaluation of Ex Priori predictions by comparison to population aggregate exposures inferred from NHANES urinary biomonitoring data
S5. Supplemental References

S1. Ex Priori model structure

S1.1. Product-specific Inputs

Where possible, Ex Priori has been harmonized with existing EPA data and modeling efforts. As such, where possible, product-specific data are gathered from EPA databases or harmonized with higher-tier EPA exposure models. Where scenario-specific product data are known or required, the user can overwrite any variable associated with a product or add and parameterize a new product.

S1.1.1. Product Composition

Product composition data are collected from CPDat [1], a large database of consumer product composition data containing over 49,000 chemicals. The full CPDat database is available for download and can be used from the EPA Chemistry Dashboard (https://www.epa.gov/chemical-research/chemical-and-products-database-cpdat). CPDat is composed of chemical use and consumer product composition data from numerous

public sources of measured, modeled, and reported data, including the Substances in Preparation in Nordic Countries (SPIN) database; information provided by companies, trade associations, and regulatory agencies such as the U.S. Environmental Protection Agency (EPA) and Food and Drug Administration (FDA); the DrugBank database of pharmaceutical products; and information mined from the Aggregated Computational Toxicology Resource (ACToR) database developed by the U.S. EPA [2]. The data are categorized by usage and function and then grouped into product categories. Ex Priori is populated with the 228 product categories used by CPDat and divided into nine broader categories: Arts and Crafts, Auto Products, Home Maintenance, Home Office, Inside the Home, Landscape/Yard, Personal Care, Pesticides, and Pet Care. Examples of the 228 CPDat product categories within each of the nine broader categories include (Arts and Crafts) Play Dough; (Auto Products) Antifreeze; (Home Maintenance) Caulk/Sealant; (Home Office) Printer Ink/Toner; (Inside the Home) Air Freshener; (Landscape/Yard) Fertilizer; (Personal Care) Body Wash; (Pesticides) Insect Repellent; and (Pet Care) Spot Cleaner. For clarity of language, Ex Priori refers to the 228 CPDat product categories as "products", envisioning each one as a single generic product (even though it likely includes multiple brands and/or varieties). The nine broader product categories are referred to as "product categories."

Ex Priori uses the CPDat average mass fraction of each chemical in each product (grams of chemical per gram of product) as the basis of its product composition data. (2) Because compositions may vary for different brands of a given product, taking the average mass fraction of each chemical across these brands may mean that the average mass fractions within a given product may sum to more or less than 100%. For example, Product X may have an average composition of 60% Solvent A, 60% Solvent B, and 60% Solvent C, summing to 180%. This is not a problem because Ex Priori considers exposures one chemical at a time and does not attempt to model chemical co-exposures. In other words, the product is not assumed to *simultaneously* consist of 60% Solvent A, 60% Solvent B, and 60% Solvent C. Rather, Ex Priori evaluates potential exposures to Solvent A for the case where Product X is 60% Solvent A; then, it evaluates potential exposures to Solvent B for the case where Product X is 60% Solvent B, etc. Therefore, average mass fractions were taken as-is, with no normalization or other transformation applied.

Product composition data are included in matrix form in the "Chem Prod Pct Matrix" tab of the Ex Priori model spreadsheet (as percentages) and are converted to mass fractions on the "Chem Prod MassFrac Matrix" tab.

S1.1.2. Habits and Practices: Mass, Frequency, and Duration of Product Use

The mass of product used during each event, frequency of use events per year, and the duration of product use for each product category are harmonized with SHEDS-HT and are presented in Isaacs, Glen [3]. Where possible, mass, frequency, and duration of product use data are drawn from the published literature. However, these data are sparse and frequently require expert judgment. This is recognized as a data need. In order to estimate an average day of exposure over the course of a year, in which products would be expected to be used intermittently, the annual frequency of product use and the mass of product used per use are multiplied together and divided by 365 to develop the average mass of product used per day (Equation S1).

Equation S1. Mass_chem

$$\mathrm{Mass}_{\mathrm{prod}} = \mathrm{Use}_{\mathrm{day}} = \mathrm{Freq}_{\mathrm{use}} \times \mathrm{Mass}_{\mathrm{product,\ per\ use}}/365 \quad \text{(S1)}$$

The mass of a given chemical in the mass of product used per day is given by multiplying the daily mass of product used by the mass fraction of chemical in the product, F_{chem} (Equation S2).

Equation S2. Mass_chem from Mass_frac_chem and Mass_prod

$$\mathrm{Mass}_{\mathrm{chem}} = \mathrm{F}_{\mathrm{chem}} \times \mathrm{Use}_{\mathrm{day}} \quad \text{(S2)}$$

Habits and practices data and calculations are performed in the "Products Input" tab of the Ex Priori model spreadsheet.

S1.1.3. Dermal and Floor Factors and Outdoor Dilution Factors

Two factors, dermal and floor factors, are used to govern how mass that is emitted during product use that does not remain in the air compartment is divided between settling on the skin or settling on the floor, where the floor is used as a proxy for interior surfaces. These factors vary based on the product and the way it is administered. For example, a lotion applied directly to the skin will have a larger dermal factor than a product used outdoors.

Two dermal default values presented in Isaacs, Glen [3] are used to estimate exposure in Ex Priori: the percent of product in contact with the skin and the percent of product retained on the skin post-washing. The percent of product retained on the skin is utilized as the dermal factor and is used to calculate dermal loading in Ex Priori rather than assigning a loading and washing efficiency because of high uncertainty in the latter variable. The mass of product that partitions onto the skin is calculated using the total mass of product used per day and the dermal factor (Equation S3).

Equation S3. Use_day_dermal

$$\mathrm{Use}_{\mathrm{day,\ dermal}} = \mathrm{Use}_{\mathrm{day}} \times \mathrm{Dermal\_factor} \quad \text{(S3)}$$

Here, we assume that for products used indoors, the percentage of mass that is available to the floor (Floor_factor) is generally determined by taking 1 - Dermal_factor. For example, if 90% of the product is in contact with the skin, 100%-90%, or 10%, is assumed to be available to the floor and other indoor surfaces. Product categories with the word "exterior" in the product name and products in the Landscape/Yard global category are assumed to be used outdoors and are therefore assigned a floor factor of zero. This assumption may underestimate the outdoor incidental ingestion exposure of product settled onto soil, particularly in cases where a large amount of exterior product would be applied to a relatively small parcel of soil, creating "hot spots" of contaminated soil that could be subsequently inadvertently ingested. However, considering the uncertainty in characterizing soil and soil contamination, the assumption is made that ingestion exposure to exterior products will be lower than to products used indoors.

The mass of product that partitions onto the floor is calculated using the total mass of product used per day and the floor factor (Equation S4).

Equation S4. Use_day_floor

$$\mathrm{Use}_{\mathrm{day,\ floor}} = \mathrm{Use}_{\mathrm{day}} \times \mathrm{Floor\_factor} \quad \text{(S4)}$$

Finally, an outdoor inhalation dilution factor ($\mathrm{Inh}_{\mathrm{dil}}$) of 20 is applied to product categories assumed to be used outdoors (those with the word "exterior" in the product name and products in the Landscape/Yard category); a dilution factor of 1 is applied to other products. The outdoor dilution factor is derived from Klepeis, Gabel [4]. This means that for inhalation exposure purposes, the effective mass of product accounting for outdoor dilution is given by Equation S5.

Equation S5. Use_day_air

$$\mathrm{Use}_{\mathrm{day,\ air}} = \mathrm{Use}_{\mathrm{day}} \;/\; \mathrm{Inh}_{\mathrm{dil}} \quad \text{(S5)}$$

Dermal and floor factor values and calculations as well as outdoor dilution calculations are performed in the "Product Inputs" tab of the Ex Priori model spreadsheet.

S1.2. Chemical-specific Inputs

The chemical-specific inputs utilized by Ex Priori include physicochemical properties (Supplemental Table S2) and quantitative structure–activity relationship (QSAR) predictions/estimates for internal exposure estimates (Supplemental Table S3). Physicochemical

properties were determined for each chemical using OPERA [5]. The property values in the current version of Ex Priori are from a September 2016 version of OPERA.

Out of 1634 chemicals included in CPDat, 1108 chemicals could be mapped to unique structures on the CCD and could therefore be assigned physicochemical properties. Therefore, only these 1108 chemicals are considered in this analysis. QSAR predictions are based on the published literature. QSAR models are discussed in detail in the Supplemental Material section for each exposure pathway in which they are used. Chemical-specific inputs can be found in the "Chemical Inputs" tab of the Ex Priori model spreadsheet.

S1.3. Receptor and Environmental Inputs

To model a human receptor, Ex Priori requires human exposure factors, including inhalation rate, skin surface area, hand-to-mouth fraction, and the ratio of hand surface area to body surface area. To model an indoor exposure scenario, Ex Priori also requires environmental exposure factors including room size, building ventilation rate, dust load per square meter of floor, air volume of near-field "user bubble", air flow between user bubble and the larger room, and air concentrations of total suspended particulates and PM2.5. These parameters are detailed in Supplemental Table S4 and can be found in the "Scenario Inputs" tab of the Ex Priori model spreadsheet.

S1.4. Exposure Pathways

The mass of each chemical and the overall product mass across the product categories used are apportioned across inhalation, dermal, and ingestion routes, as shown in Figure S2 in the main text.

First, the chemical mass is treated as partitioning between the product itself and the air in the room based on the air–water partitioning coefficient. The mass that partitions to the air is initially assumed to be available to the inhalation pathway, and the remainder is available to the floor and skin surface, as determined by the product-specific floor and dermal factors. The chemical mass that partitions via the air compartment can be inhaled either during or post-use. Of the chemical deposited on the skin, chemical mass can be absorbed dermally, as determined by the chemical-specific dermal flux, or mass can be ingested by hand-to-mouth contact. Of the chemical that is deposited on the floor, mass can be ingested via incidental dust ingestion facilitated by hand- or object-to-mouth contact, or the deposited dust can be resuspended. Small resuspended particles can be inhaled deep into the lungs, while larger particles can be inhaled, trapped in the upper airway, and subsequently ingested. Exposure-by-pathway is discussed in finer detail in the following sections.

S1.4.1. Inhalation

Inhalation exposure is broken into three pathways: direct airborne exposure during use, indirect airborne exposure post-use, and indirect inhalation of resuspended mass originally deposited on the floor. The calculations for these three inhalation pathways can be found in the "Inh_in_use", "Inh_after_use", and "Inh_particle" tabs of the Ex Priori spreadsheet, respectively.

S1.4.1.1. Preliminaries

Direct and indirect airborne exposures are modeled using a two-zone model [6], which assumes a smaller volume of air constituting a near-field "user bubble" within a larger room. The volume of the user bubble is denoted as $V_{bubble,}$ and that of the larger room is denoted as V_{room}. Air is exchanged between the user bubble and a larger room at flow rate β (m^3/hour). The air exchange rate of the larger room (building ventilation) is denoted as AER (number of air changes per hour), which converts to flow rate Q = AER × V_{room} (m^3/hour). The emission rate G of a specified chemical from the product into the air is assumed to be constant during product use and zero after product use. Other relevant parameters include the average adult inhalation rate (Inh_{rate}, m^3/day) from the Exposure

Factors Handbook [7] and the duration of product use (Dur_{use}, minutes, found on the worksheet "Product Inputs"). For compactness of notation, it is convenient to convert the duration of use into units of hours and to refer to it as D (Equation S6). (Note that the column "Dur_use" on the worksheets "Inh_in_use", "Inh_after_use", and "Inh_total" remains in units of minutes; the conversion to hours occurs in each formula that uses this quantity.)

Equation S6. Dur_use converted to hours

$$D = \text{Dur\_use}/60 \tag{S6}$$

S1.4.1.2. Approximation of Constant Emission Rate G

The constant emission rate G is approximated using the chemical-specific air–water partitioning coefficient K_{aw}. K_{aw} is a physicochemical property that is known (either measured or QSAR-predicted) for the chemicals considered by Ex Priori.

Imagine a volume of liquid product, V_{liq}, that contains a mass of chemical $\text{Mass}_{\text{chem}}$. The product is allowed to stand in contact with a volume of air, V_{air}. The chemical will diffuse from the liquid into the air. If the diffusion is allowed to proceed for a long-enough time, then the chemical concentrations in the air and liquid ($C_{\text{chem-air}}$ and $C_{\text{chem-liq}}$) will reach steady state. At steady state, the mass of chemical in the air is denoted as $\text{Mass}_{\text{chem-air}}$,. and the mass of the chemical remaining in the liquid product is denoted $\text{Mass}_{\text{chem-liq}}$. The steady-state ratio of $C_{\text{chem-air}}$ to $C_{\text{chem-liq}}$ is described by K_{aw} (Equation S7).

Equation S7. Kaw as ratio C_chem-air/C_chem-liq

$$K_{aw} = \frac{C_{\text{chem-air}}}{C_{\text{chem-liq}}} = \frac{\text{Mass}_{\text{chem-air}}/V_{\text{air}}}{\text{Mass}_{\text{chem-liq}}/V_{\text{liq}}} \tag{S7}$$

Additionally, at steady state, the total mass of chemical originally in the product is divided between $\text{Mass}_{\text{chem-air}}$ and $\text{Mass}_{\text{chem-liq}}$ (Equation S8).

Equation S8. Mass balance between air and liquid

$$\text{Mass}_{\text{chem}} = \text{Mass}_{\text{chem-air}} + \text{Mass}_{\text{chem-liq}} \tag{S8}$$

If we can use K_{aw} to estimate a value for $\text{Mass}_{\text{chem-air}}$, then we can estimate the constant emission rate G by assuming that $\text{Mass}_{\text{chem-air}}$ is emitted steadily over the duration of product use (Equation S9).

Equation S9. Constant emission rate

$$G = \frac{\text{Mass}_{\text{chem-air}}}{D} \tag{S9}$$

However, in order to use K_{aw} to estimate a value for $\text{Mass}_{\text{chem-air}}$ and therefore G, we need to know not only K_{aw}, but also V_{liq}, V_{air}, and $\text{Mass}_{\text{chem-liq}}$ (based on Equation S7).

To find V_{liq}, assume that the product is aqueous (liquid) and has a density ρ_{prod} equal to water (1 g/cm^3) and a mass denoted by $\text{Mass}_{\text{prod}}$. This assumption means that Ex Priori is implicitly limited in its scope to aqueous consumer products and is not applicable to solid consumer articles. Then, V_{liq} (in units of m^3) is given by Equation S10, where the factor 1×10^{-6} converts the liquid volume from cm^3 to m^3.

Equation S10. Vliq from Mass_prod

$$V_{\text{liq}} = \frac{\text{Mass}_{\text{prod}}}{\rho_{\text{prod}}} \times 1 \times 10^{-6} \tag{S10}$$

To find V_{air}, consider that under the assumptions of the two-zone model, the liquid product stands in contact with the air inside the user bubble. However, air is continuously flowing through the user bubble while the product is being used, so ultimately, the chemical will diffuse into a larger volume of air than the fixed volume of the user bubble. In fact, the chemical will diffuse into the total volume of air that flows through the user bubble during product use. Under the assumptions of the two-zone model, the air flow rate through the user bubble is β (m^3/hour). The duration of product use was defined to be D

hours. Therefore, the total volume of air that flows through the bubble during product use is given by Equation S11.

Equation S11. V_air = beta*D

$$V_{air} = \beta D \tag{S11}$$

In other words, we assume that the total amount of chemical emitted will be the same as if the product stood in contact with the full volume of air βD all at once and long enough for diffusion to reach steady state. Of course, in reality, diffusion (and therefore the emission rate) is time-dependent; however, we make this simplifying assumption for reasons of computational tractability. See Section S3.3 for further discussion and some analysis of potential errors in this assumption.

Substituting the expressions for V_{liq} and V_{air} (Equation S10 and Equation S11, respectively) into the definition of K_{aw} (Equation S7), the following equation emerges (Equation S12):

Equation S12. Kaw with definitions of Vair and Vliq

$$K_{aw} = \frac{C_{\text{chem-air}}}{C_{\text{chem-liq}}} = \frac{\text{Mass}_{\text{chem-air}}/\beta D}{\text{Mass}_{\text{chem-liq}}/\left(\text{Mass}_{\text{prod}}/\rho_{\text{prod}} \times 1 \times 10^{-6}\right)} \tag{S12}$$

Finally, to find $\text{Mass}_{\text{chem-liq}}$, note that at steady state, the total mass of chemical in the product is divided between the air and the liquid (Equation S13).

Equation S13. Mass balance between air and liquid

$$\text{Mass}_{\text{chem}} = \text{Mass}_{\text{chem-air}} + \text{Mass}_{\text{chem-liq}} \tag{S13}$$

Then, combining Equation S12 and Equation S13, $\text{Mass}_{\text{chem-air}}$ is given by Equation S14.

Equation S14. Expression for Mass_chem-air

$$\text{Mass}_{\text{chem-air}} = \frac{K_{aw} \times \text{Mass}_{\text{chem}} \times \beta D}{\frac{\text{Mass}_{\text{prod}}}{\rho_{\text{prod}}} + K_{aw} \times \beta D} \tag{S14}$$

Equation S14 implies that the air concentration $C_{\text{chem-air}}$ is given by Equation S15.

Equation S15. C_chem-air

$$C_{\text{chem-air}} = \text{Mass}_{\text{chem-air}}/\beta D \tag{S15}$$

Because this steady-state emissions model is only an approximation, it is possible that it may predict a value for $\text{Mass}_{\text{chem-air}}$ that would produce an air concentration higher than the chemical's theoretical saturation concentration in air. To correct for this possibility, the theoretical air saturation concentration is predicted using a QSAR model (Equation S16).

Equation S16. Csat QSAR

$$C_{sat} = \frac{133 \times 0.0075 \times \text{VP} \times \text{MW}}{8.314 \times 298} \tag{S16}$$

The fraction of total mass that goes into the air is termed In_{air} (Equation S17).

Equation S17. In_air

$$C_{sat} = \frac{133 \times 0.0075 \times \text{VP} \times \text{MW}}{8.314 \times 298} \tag{S17}$$

The remainder of the mass (1- In_{air}) is available to the skin and the floor and is divided between the two compartments using the dermal and floor factors.

Equation S14 describes the mass of chemical (and with Equation S9, the emissions rate) for a chemical in a single product. Rather than calculating inhalation exposure separately for each product and then summing, first, product masses and chemical masses are combined (as though all products that contained a given chemical were mixed together and used all at once), and then inhalation exposure is calculated only once, for the combined product.

To combine product masses, $\text{Mass}_{\text{prod}}$ in Equation S14 is replaced by $\text{Sum}_{\text{prod\_cat}}$, a weighted sum of product masses across product categories (Equation S18). The weights are the product-category weights, where W_j is the weight for the jth product category. Within each product category, the $\text{Use}_{\text{day, air}}$ values (Equation S5) are summed over all products in the category (including any outdoor dilution factors). This calculation is performed in tab "Sum Prod Cat" of the Ex Priori model spreadsheet.

Equation S18. Sum_prod_cat

$$\text{Sum}_{\text{prod\_cat}} = \sum_{\text{j} \in \text{product categories}} W_j \times \left(\sum_{\substack{\text{I} \in \text{products in category } j \\ \text{containing specified chemical}}} \text{Use}_{\text{day, air},i} \right) \quad \text{(S18)}$$

To combine chemical mass across products, for a given chemical, $\text{Mass}_{\text{chem}}$ in Equation S14 and Equation S17 is replaced by $\text{Sum}_{\text{chem\_cat\_inh}}$, a weighted sum of chemical masses across product categories (Equation S19). The weights are the product-category weights, where W_j is the weight for the jth product category. Here, the effective chemical mass (including any outdoor dilution factor) is calculated for each product by multiplying the $\text{Use}_{\text{day, air}}$ values (Equation S5) for each product by the mass fraction of chemical in the product, $F_{\text{chem},i}$. This calculation is performed in the "Mass Chem Air" tab of the Ex Priori model spreadsheet.

Equation S19. Sum_chem_chat_inh

$$\text{Sum}_{\text{chem\_cat\_inh}} = \sum_{\text{j} \in \text{product categories}} W_j \times \left(\sum_{i \in \text{products in category } j} \text{Use}_{\text{day, air},i} \times F_{\text{chem},i} \right) \quad \text{(S19)}$$

Finally, the duration of use, Dur_use, is different for every product. When products are combined, D in Equation S14 (where D = Dur_use/60) is replaced by $\text{Dur\_use}_{\text{avg}}/60$, where $\text{Dur\_use}_{\text{avg}}$ is the duration of use averaged across all products that contain a given chemical (Equation S20). This calculation is performed in the "Prod Dur" tab of the Ex Priori model spreadsheet.

Equation S20. Dur_use_avg

$$\text{Dur\_use}_{\text{avg}} = \frac{1}{\text{\# products containing specified chemical}} \sum_{\substack{i \in \text{all products} \\ \text{containing specified chemical}}} \text{Dur\_use}_i \quad \text{(S20)}$$

S1.4.1.3. Airborne Direct (Gases and Particles)

Under the assumptions of the two-zone model described above, it can be shown that inhalation exposure during product use ($\text{Intake}_{\text{inh\_in\_use}}$) is given by Equation S21–Equation S23 (full derivation available in Section S3.1). These calculations are performed in the "Inh_in_use" tab of the Ex Priori model spreadsheet.

Equation S21. Intake_inh_in_use

$$\text{Intake}_{\text{inh\_in\_use}} = \text{Inh\_rate}/24 \times \left\{ \frac{\alpha_1}{\lambda_1} [\exp(\lambda_1 D) - 1] + \frac{\alpha_2}{\lambda_2} [\exp(\lambda_2 D) - 1] + \alpha_3 [D] \right\} \quad \text{(S21)}$$

Equation S22. Coefficients for two-zone model (lambdas, a, b, c, d)

$$\begin{gathered} \lambda_2 = \frac{-(a+d) - \sqrt{(a+d)^2 - 4(ad - ac)}}{2} \\ a = \beta / V_{\text{bubble}} \\ b = 1/V_{\text{bubble}} \\ c = \beta / V_{\text{room}} \\ d = (Q + \beta)/V_{\text{room}} \end{gathered} \quad \text{(S22)}$$

Equation S23. Alpha coefficients for Intake_inh_in_use

$$\alpha_1 = G\left(\frac{\beta Q + \lambda_2 V_{\text{bubble}}(\beta + Q)}{\beta Q V_{\text{bubble}}(\lambda_1 - \lambda_2)}\right)$$
$$\alpha_2 = -G\left(\frac{\beta Q + \lambda_1 V_{\text{bubble}}(\beta + Q)}{\beta Q V_{\text{bubble}}(\lambda_1 - \lambda_2)}\right) \quad \text{(S23)}$$
$$\alpha_3 = \frac{G}{\left(\frac{\beta}{\beta + Q}\right)Q}$$

S1.4.1.4. Airborne Indirect (Gases)

After product use, the airborne concentration of the chemical of interest will not immediately return to zero (or background). Instead, the chemical will be removed via ventilation, reaction, or sorption. This screening tool only considers removal by ventilation in the context of the two-zone model. It can be shown that under the two-zone model assumptions above, the inhalation intake after product use (Intake_inh_after_use) (for the remainder of a 24-hour period after product use duration D) is given by Equation S24–Equation S25. (Full derivations are provided in Section S3.2.) These calculations are performed in the "Inh_after_use" tab of the Ex Priori model spreadsheet.

Equation S24. Intake_inh_after_use

$$\text{Intake\_inh\_after\_use} = \text{Inh\_rate}/24 \times \left\{\frac{\delta_1}{\lambda_1}\left[\exp\left(\lambda_1(24 - D)\right) - 1\right] + \frac{\delta_2}{\lambda_2}\left[\exp\left(\lambda_2(24 - D)\right) - 1\right]\right\} \quad \text{(S24)}$$

Equation S25. Delta coefficients for Intake_inh_after_use

$$\delta_1 = \alpha_1 \exp(\lambda_1 D) + \alpha_3\left[1 - \left(\frac{1}{\lambda_2 - \lambda_1}\right)\left(\frac{a(c-d)}{d} - \lambda_1\right)\right]$$
$$\delta_2 = \alpha_2 \exp(\lambda_2 D) + \left(\frac{\alpha_3}{\lambda_2 - \lambda_1}\right)\left[\frac{a(c-d)}{d} - \lambda_1\right] \quad \text{(S25)}$$

(α_1, α_2, and α_3 are given by Equation S23, and λ_1, λ_2, and a are given by Equation S22.)

S1.4.1.5. Airborne Indirect (Particles)

Chemical mass that falls to the floor after use mixes with the dust on the floor and can then become resuspended and inhaled. Resuspension rates are highly variable depending on particle size and activity within the room. To estimate the inhalation of resuspended particles, first, the total chemical mass available to be deposited on the floor ($\text{Sum}_{\text{chem\_cat\_floor}}$, Equation S26) is estimated as $\text{Use}_{\text{day, floor}}$ multiplied by the mass fraction of the chemical in each product (F_{chem}), which is summed first within a product category, and then a weighted sum is taken over all product categories using the product category weights (where W_j is the weight for the jth product category). These calculations are performed in the "Sum_chem_cat_floor" tab of the Ex Priori model spreadsheet.

Equation S26. Sum_chem_cat_floor

$$\text{Sum}_{\text{chem\_cat\_floor}} = \sum_{j \in \text{product categories}} W_j \times \left(\sum_{\substack{i \in \text{products in category } j \\ \text{containing specified chemical}}} \text{Use}_{\text{day, floor}, i} \times F_{\text{chem},i}\right) \quad \text{(S26)}$$

The chemical available to the floor ($\text{Sum}_{\text{chem\_cat\_floor}}$) is subject to air partitioning, so the final mass of chemical on the floor ($\text{Chem}_{\text{floor}}$) is equal to $\text{Sum}_{\text{chem\_cat\_floor}}$ multiplied by the fraction of chemical that does *not* partition into the air (Equation S27).

Equation S27. Chem_floor

$$\text{Chem}_{\text{floor}} = \text{Sum}_{\text{chem\_cat\_floor}} \times (1 - \text{In}_{\text{air}}) \quad \text{(S27)}$$

The mass of dust on the floor ($Dust_{floor\_mass}$) is given by the dust floor loading (particles per unit area) multiplied by the floor surface area (Equation S28). ($Dust_{floor\_load}$ levels are from Wilson, Jones-Otazo [8].)

Equation S28. Dust_floor_load

$$\mathrm{Dust_{floor\_mass}} = \mathrm{Dust_{floor\_load}} \times \mathrm{room_l} \times \mathrm{room_w} \quad \text{(S28)}$$

The mass of chemical on the floor $\mathrm{Chem_{floor}}$ is then assumed to mix with the dust available on the floor, resulting in a mass fraction of dust that is composed of the chemical of interest, $\mathrm{Chem_{dust\_frac}}$ (Equation S29).

Equation S29. Chem_dust_frac

$$\mathrm{Chem_{dust\_frac}} = \frac{\mathrm{Chem_{floor}}}{\mathrm{Dust_{floor\_mass}} + \mathrm{Chem_{floor}}} \quad \text{(S29)}$$

Particle inhalation exposure was assumed to involve dust particles less than 2.5 microns in diameter ($PM_{2.5}$), which can penetrate deeply into the lung. The concentration of $PM_{2.5}$ ($C_{PM2.5}$) was taken from Deshpande, Frey [9]. For this estimation, the settled dust and $PM_{2.5}$ were assumed to be in equilibrium with respect to the chemical of interest. The exposure from particle inhalation ($\mathrm{Amt_{inh\_particle}}$) can then be calculated (Equation S30). These calculations are performed in the "Inh_particle" tab of the Ex Priori model spreadsheet.

Equation S30. Amt_inh_particle

$$\mathrm{Amt_{inh\_particle}} = \mathrm{Inh_{rate}} \times \mathrm{Chem_{dust\_frac}} \times C_{PM2.5} \times 1\ \mathrm{day} \quad \text{(S30)}$$

S1.4.1.6. Inhalation Absorption

The mass of inhaled chemical that is subsequently absorbed (Abs_{inh}) is calculated using the blood:air partitioning coefficient (K_{ba}) estimated using the methodology from Buist, Wit-Bos [10] (Equation S31).

Equation S31. Log10 K_ba

$$\log_{10}(K_{ba}) = 6.96 - 1.04 \times \log_{10}(VP) - 0.533 \times \log_{10}(K_{ow}) - 0.00495 \times MW \quad \text{(S31)}$$

The K_{ba} is used to calculate the fraction of the total chemical mass inhaled that will partition to the blood (Abs_{frac_inh}) (Equation S32).

Equation S32. Abs_frac_inh

$$\mathrm{Abs_{frac\_inh}} = 1 - \frac{K_{ba}}{1 + K_{ba}} \quad \text{(S32)}$$

This is multiplied by the total mass inhaled to obtain the mass absorbed (Equation S33). These calculations are performed in the "Inh_total" tab of the Ex Priori model spreadsheet.

Equation S33. Abs_inh

$$\mathrm{Abs_{inh}} = \mathrm{Abs_{frac\_inh}} \times (\mathrm{Intake_{inh\_in\_use}} + \mathrm{Intake_{inh\_after\_use}} + \mathrm{Amt_{inh\_particle}}) \quad \text{(S33)}$$

S1.4.2. Dermal Direct

Dermal exposure (calculated in the "Dermal" tab of the Ex Priori model spreadsheet) is described by the steady-state flux of chemical through the skin (JSS) and the surface area of skin exposure (SA_{skin}). To estimate this, first, the total chemical mass available to be deposited on the skin ($Sum_{chem\_cat\_dermal}$, Equation S34) is estimated as Use_{day_dermal}, multiplied by the mass fraction of chemical within a product F_{chem}, and summed first over products within a product category, and then a weighted sum is taken over all product categories using the product category weights (W_j is the weight for the j^{th-} product category).

Equation S34. Sum_chem_cat_dermal

$$\text{Sum}_{\text{chem\_cat\_dermal}} = \sum_{j \in \text{product categories}} W_j \times \left(\sum_{i \in \text{products in category } j} \text{Use}_{\text{day, dermal },i} \times F_{\text{chem},i} \right) \quad \text{(S34)}$$

As shown in Equation S35–Equation S44, the chemical mass fluxed through the skin ($\text{Abs}_{\text{dermal-flux}}$) in units of g/day is a function of molecular weight and octanol–water partitioning, as presented in Weschler and Nazaroff [11]. The thickness of the skin lipid layer, Th_l, is assumed to be equal to 1.3 microns [11]. The default area of skin exposure is set equal to SA_{adult}, the surface area of an adult human as reported in the Exposure Factors Handbook [7]. Because the flux rate is independent of the chemical loading on the skin, an upper bound of dermal absorption was set to ensure that the mass absorbed was not greater than the total amount on the skin ($\text{Skin}_{\text{load}} \times \text{SA}_{\text{adult}}$). The skin loading per unit surface area was equal to the mass of chemical available to the skin, estimated as the fraction of chemical not in the air (1-In_{air}) multiplied by the mass of chemical available to the skin ($\text{Sum}_{\text{chem\_cat\_dermal}}$).

In other words, the model assumes that out of the chemical available to the skin (defined by $\text{Sum}_{\text{chem\_cat\_dermal}}$), some fraction In_{air} partitions into the air before any dermal absorption occurs. In_{air} is defined according to the chemical air emissions model described in the Inhalation section (Equation S17). Therefore, assumptions that affect chemical air emissions will also indirectly affect dermal exposure.

Equation S35. Abs_dermal

$$\text{Abs}_{\text{dermal}} = \min[\text{Skin}_{\text{load}} \times \text{SA}_{\text{adult}}, \text{Abs}_{\text{dermal-flux}}] \quad \text{(S35)}$$

Equation S36. Abs_dermal_flux

$$\text{Abs}_{\text{dermal-flux}} = JSS \times \text{SA}_{\text{adult}} \quad \text{(S36)}$$

Equation S37. JSS

$$JSS = k_{p_l} \times C_l \quad \text{(S37)}$$

Equation S38. k_pl

$$k_{p_l} = \frac{k_{p_w}}{K_{lw}} \quad \text{(S38)}$$

Equation S39. C_l

$$C_l = \frac{\text{Skin}_{\text{load}}}{Th_l} \quad \text{(S39)}$$

Equation S40. Skin_load

$$\text{Skin}_{\text{load}} = (1 - \text{In}_{\text{air}}) \times \frac{\text{Sum}_{\text{chem\_cat\_dermal}}}{\text{SA}_{\text{adult}}} \quad \text{(S40)}$$

Equation S41. Log K_bw

$$log(K_{lw}) = 0.74 \times log(K_{ow}) \quad \text{(S41)}$$

Equation S42. k_p-w

$$k_{p\_w} = \frac{k_{p\_cw}}{1 + B} \quad \text{(S42)}$$

Equation S43. B

$$B = \frac{k_{p\_cw} \times MW^{0.5}}{2.6} \quad \text{(S43)}$$

Equation S44. Log k_p_cw

$$log(k_{p\_cw}) = 0.7 \times log(K_{ow}) - 0.0722 \times MW^{\frac{2}{3}} - 5.252 \quad \text{(S44)}$$

Note that $\text{Use}_{\text{day, dermal},i}$ includes the dermal factor for product i, which represents the fraction of product retained on the skin after washing, as derived in SHEDS-HT. There is no further wash-off or other pathway to remove product from the skin; rather, the post-wash-off product fraction is assumed to go onto the skin and to stay on the skin. This is a conservative assumption, but for prioritization purposes, it may still produce useful results.

S1.4.3. Ingestion

Ingestion exposure in Ex Priori is broken into three pathways: indirect ingestion of settled dust, indirect ingestion of resuspended mass originally deposited on the floor, and indirect ingestion from hand-to-mouth contact. Direct ingestion was not included in the model. None of the products currently included in Ex Priori are intended to be ingested. Unintentional ingestion would require additional data to model: ingestion rates of specific products are highly uncertain and are expected to be vary widely in the population. Additionally, Ex Priori does not include pathways that are more commonly associated with consumer article exposure, rather than with consumer product exposure. These include the air-to-skin dermal pathway and ingestion via transfer from food packaging to food.

The results in the "Ingestion" tab of the Ex Priori model spreadsheet are drawn from the equations in the following sections. All of these depend upon the quantity $\text{Chem}_{\text{dust\_frac}}$ (Equation S29), which, in turn, depends on the quantity $\text{Chem}_{\text{floor}}$ (Equation S27), estimated as the fraction of chemical that does not partition to the air (1-In_{air}) multiplied by the mass of chemical available to the floor ($\text{Sum}_{\text{chem\_cat\_floor}}$).

In other words, the model assumes that out of the chemical available to the floor (defined by $\text{Sum}_{\text{chem\_cat\_floor}}$), some fraction In_{air} partitions into the air before any chemical reaches the floor, reducing the final mass of chemical on the floor. In_{air} is defined according to the chemical air emissions model described in the Inhalation section (Equation S17). Therefore, assumptions that affect chemical air emissions will also indirectly affect ingestion exposure.

S1.4.3.1. Ingestion Indirect (Incidental)

The Exposure Factors Handbook reports that an adult ingests 30 mg/day of house dust due to incidental (i.e., not purposeful) ingestion (5). This daily amount of dust ingestion ($\text{Dust}_{\text{ing\_rate}}$) is multiplied by the mass fraction of the chemical that partitions household dust ($\text{Chem}_{\text{dust\_frac}}$) to arrive at the daily incidental chemical ingestion from dust ($\text{Intake}_{\text{ing\_floor}}$).

Equation S45. Amt_ing_floor

$$\text{Intake}_{\text{ing\_floor}} = \text{Dust}_{\text{ing\_rate}} \times \text{Chem}_{\text{dust\_frac}} \quad \text{(S45)}$$

S1.4.3.2. Ingestion Indirect (Hand-to-mouth)

In addition to absorption of a chemical through the skin, a chemical deposited on the skin can be ingested due to hand-to-mouth contact. This is included in Ex Priori as the amount of a chemical ingested due to hand-to-mouth ($\text{Intake}_{\text{ing\_dermal}}$, Equation S46), which is a function of the amount of chemical remaining on the skin available for oral ingestion ($\text{Avail}_{\text{oral}}$, Equation S47) after considering the removal pathway of absorption as well as the fraction of the body ($\text{SA}_{\text{hand-body}}$) that is available for hand-to-mouth contact and the hand to mouth transfer rate ($\text{Frac}_{\text{hand\_mouth}}$), as used in SHEDS-Multi Media [12]. The $\text{SA}_{\text{hand-body}}$ is the surface area of the hands divided by the body in the current version of Ex Priori; however, this value can be adjusted by the user.

Equation S46. Intake_ing_dermal

$$\text{Intake}_{\text{ing\_dermal}} = \text{Avail}_{\text{oral}} \times \text{SA}_{\text{hand\_body}} \times \text{Frac}_{\text{hand\_mouth}} \quad \text{(S46)}$$

Equation S47. Avail_oral

$$\text{Avail}_{\text{oral}} = \text{Skin}_{\text{load}} - \text{Abs}_{\text{dermal}} \tag{S47}$$

(See Equation S40 for $\text{Skin}_{\text{load}}$ and Equation S35 for $\text{Abs}_{\text{dermal}}$.)

S1.4.3.3. Ingestion Indirect (Surface Resuspension)

Chemical mass that falls to the floor and is subsequently resuspended can be in the form of small, inhalable particles or in the form of larger particles that do not penetrate deeply within the lung. When these larger particles are inhaled, they are caught in the upper airway, "coughed up", and then swallowed, resulting in ingestion exposure. Exposure via this pathway (Equation S48) is calculated similarly to the airborne indirect particle pathway (Equation S30), with the exception that a typical indoor total suspended particulate (TSP) concentration (C_{TSP}) is used in place of a $PM_{2.5}$ concentration.

Equation S48. Intake_ing_particle

$$\text{Intake}_{\text{ing\_particle}} = \text{Inh}_{\text{rate}} \times \text{Chem}_{\text{dust frac}} \times C_{TSP} \times 1\text{ day} \tag{S48}$$

S1.4.3.4. Ingestion Absorption

The mass of the chemical ingested that is subsequently absorbed (Abs_{Ing}, Equation S50) is calculated by multiplying the ingestion absorption fraction (Frac_{Ing}, Equation S51) by the total mass of chemical ingested through all routes described in the preceding sections (Equation S49). The oral absorption rate constant is calculated as a function of the octanol–water partition coefficient (K_{ow}) and the polar surface area (PSA) (Equation S52). Note that Equation S52 is based on a model [13] and was originally formulated using $\log_{10} D_{6.0}$—the octanol–water distribution coefficient at pH 6.0—rather than $\log_{10} K_{ow}$, the octanol–water partition coefficient for neutral compounds. The two are equivalent for non-ionizable compounds but may be different for ionizable compounds.

Equation S49. Intake_ing_total

$$\text{Intake}_{\text{ing\_total}} = \text{Intake}_{\text{ing\_floor}} + \text{Intake}_{\text{ing\_particle}} + \text{Intake}_{\text{ing\_dermal}} \tag{S49}$$

Equation S50. Abs_ing

$$\text{Abs}_{\text{Ing}} = \text{Frac}_{\text{Ing}} \times \text{Intake}_{\text{ing\_total}} \tag{S50}$$

Equation S51. Frac_ing

$$\text{Frac}_{\text{Ing}} = 1 - (1 + 0.32k_a)^{-7} \tag{S51}$$

Equation S52. Log k_a

$$\log_{10}(k_a) = 0.623 + 0.154 \times \log_{10}(K_{ow}) - 0.007 \times PSA \tag{S52}$$

S1.5. Estimation of Internal Dose

The total absorbed dose is calculated as a weighted sum of absorbed doses from each route using the route weights, where W_{inh} is the weight for the inhalation route, W_{ing} is the weight for the ingestion route, and W_{dermal} is the weight for the dermal route.

To calculate the dose remaining in the body after 24 hours (ADME_{rem}), the estimated toxicokinetic clearance rate of each chemical is approximated by calculating a chemical-specific half-life in the body (Equation S53). The half-lives were estimated as a function of the octanol–water partitioning coefficient based on a previously published regression relation [14] (Equation S54).

Equation S53. ADME_rem

$$\text{ADME}_{\text{rem}} = (W_{\text{inh}}\text{Abs}_{\text{inh}} + W_{\text{ing}}\text{Abs}_{\text{ing}} + W_{\text{dermal}}\text{Abs}_{\text{dermal}})/2\text{^}\{24/t_{1/2}\} \tag{S53}$$

Equation S54. ADME half-life

$$\log_{10} t_{1/2} = 0.427 + 0.288 \log_{10} K_{ow} \quad \text{(S54)}$$

$ADME_{rem}$ serves as the final output of the model and is calculated in the "ADME" tab of Ex Priori.

S1.6. Chemical Rankings

Chemicals are ranked from highest to lowest $ADME_{rem}$ in the "Outputs" tab in the Ex Priori model spreadsheet. Any ties are broken randomly by adding a random number between 0 and 1 to each rank number. The tie-breaking process is performed in the tab "Output Visualizations" of the Ex Priori model spreadsheet and is so named because it is required for the visualizations in the "Dashboard" tab.

S2. Tables of Ex Priori model inputs and calculated variables

Table S1. Product-specific inputs used by Ex Priori. Values are product-specific. Values for each product may be found in the "Product Inputs" tab of the Ex Priori spreadsheet.

Parameter	Units	Description
Freq_use	# uses/year	Frequency of product use
Mass_prod	g/use	Mass of product used per event
Dur_use	min	Duration of product use
Type_use		Optional variable to assign dermal and floor factors
Dermal_factor	%	The mass percentage of product that goes onto the skin.
Floor_factor	%	The mass percentage of product that goes onto the floor.
P_{prod}	g/cm^3	Density of liquid products. By default, assumed to be 1 for all products.

Table S2. Chemical-specific input parameters used by Ex Priori. Values are chemical-specific. Values for each chemical may be found in the "Chemical Inputs" tab of the Ex Priori spreadsheet.

Parameter	Units	Description
MW	g/mol	Molecular weight
Kaw	Unitless	Air–water partition coefficient
VP	Pa	Vapor pressure
$\text{Log}_{10}\ K_{ow}$	Unitless ($\log_{10}$)	Log octanol–water coefficient
**PSA**	Å^2	Polar surface area of molecule

Table S3. Chemical-specific parameters calculated by Ex Priori. Values are chemical-specific. Values for each chemical may be found in the "Chemical Inputs" tab of the Ex Priori spreadsheet.

Parameter	Units	Description	Formula	Source
K_{ba}	Unitless	Blood:air partition coefficient	6.96−1.04*$\log_{10}$ VP 0.533*$\log_{10}$ K_{ow} −0.00495*MW	Buist, Wit-Bos [10]
C_{sat}	g/m^3	Saturation concentration in air	(VP*0.0075)*MW/(8.314*298)*133	United States Environmental Protection Agency [15]
Abs_frac_inh	unitless	Absorption fraction for the inhalation intake pathway based on blood:air partitioning	1−(K_{ab}/(K_{ab} + 1))	Derived
$K_{p\text{-}cw}$	m/hr	Permeability coefficient through stratum corneum of an SVOC when the species concentration is measured in water in contact with skin	10^(0.7* $\log_{10}$ K_{ow} -0.0722*(MW^(2/3))-5.252)*3600/100	Weschler and Nazaroff [11]
B	unitless	Ratio of the stratum corneum permeability to the viable epidermis permeability	($K_{p\text{-}cw}$ *MW^0.5)/2.6	Weschler and Nazaroff [11]

k_{lw}	unitless	Coefficient of equilibrium partitioning for an SVOC between skin-surface pipis and water	$K_{p\text{-}cw}/(1+B)$	Weschler and Nazaroff [11]
k_{pw}	m/hr	Permeability coefficient through the stratum corneum/viable epidermis composite of SVOC when the species concentration is measured in water in contact with skin	$= k_{p\text{-}cw} /(1+B)$	Weschler and Nazaroff [11]
k_{pl}	m/hr	Permeability coefficient that describes transport of SVOC dissolved in skin-surface lipids through stratum corneum/viable epidermis composite	k_{pw}/k_{lw}	Weschler and Nazaroff [11]
k_{ing}	1/hr	Oral absorption rate constant	$10^{\wedge}(0.623+0.154^{*} \log_{10} K_{ow} - 0.007^{*}PSA)$	Linnankoski, Mäkelä [13]
Abs_frac_ing	unitless	Absorption fraction for ingestion intake	$1-(1+0.32^{*}k_{ing})^{-7}$	Linnankoski, Mäkelä [13]
$t_{1/2}$	hr	Half-life of chemical in body	$10^{\wedge}(0.452 + 0.288^{*} \log_{10} K_{ow})$	Sarver, White [14]

Table S4. Environmental/receptor input variables used by Ex Priori and their default values. Values may be found in the "Scenario Inputs" tab of the Ex Priori spreadsheet.

Variable	Value	Unit	Description	Source
AER	0.45	# changes/hour	Building air exchange rate	United States Environmental Protection Agency [15]
β	82.008	m^3/hour	Air flow rate between user bubble and larger room	United States Environmental Protection Agency [15]
C_PM2.5	7.16	ug/m3	Background indoor PM2.5 concentration	Deshpande, Frey [9]
C_TSP	75	ug/m3	Background indoor PM10 concentration	Assumed
Dust_floor_load	0.52	g/m2	Mass of dust on the floor / unit area	Wilson, Jones-Otazo [8]
Dust_floor_mass	17.4928	g	Total mass of dust on the floor	Calculated: Dust_floor_load * room_l * room_w
Dust_ing_rate	30	mg	Mass of dust ingested per day	United States Environmental Protection Agency [7]
Frac_hand_mouth	0.2	unitless	Fraction of chemical that is transferred from hand to mouth	Ozkaynak, Xue [16]
Inh_dil	0.05	unitless	Dilution factor to account for increased ventilation and decreased exposure when using a product outdoors	Estimated based on Table 3 of Klepeis, Gabel [4]
Inh_rate	16.2	m^3/day	Volumetric breathing rate	United States Environmental Protection Agency [7], average of Table 6-2 Light intensity values for age groups 21+ and Table 6-1 Age groups 21+
Q	36.9	m^3/hour	Air flow rate of building ventilation	Calculated: AER * V_room
Room_l, room_w	5.80	m	Room length and width	United States Environmental Protection Agency [7], average home volume (equivalent to 6 rooms) = 492 m3
Room_h	2.44	m	Room height	Assumed
SA_Adult	1.95	m2	Surface area of adult human	United States Environmental Protection Agency [7], Table 7-9 average of age groups 21+
SA_Hand_Body	0.05	unitless	Ratio of surface area of adult human hand to whole body	United States Environmental Protection Agency [7], average for adult
Th_l	1.3×10^{-6}	m3/m2	Thickness of skin surface lipids	Weschler and Nazaroff [11]
V_bubble	0.2	m3	Near-field volume during product use (user "bubble" compared to room volume)	United States Environmental Protection Agency [15]
V_room	82.08	m3	Room volume	Calculated: Room_l * Room_w * Room_h

Table S5. Variables calculated by Ex Priori.

Variable	Unit	Description
Abs_dermal	g/day	Dermal absorbed dose
Abs_dermal_max	g/day	Steady state flux of chemical through skin (JSS * skin area) set as an upper bound for daily dermal absorption
Abs_ing	g/day	Total absorbed ingestion dose
Abs_ing_dermal	g/day	Ingestion: hand-to-mouth (indirect) absorbed dose
Abs_ing_floor	g/day	Ingestion: dust-from-floor (indirect) absorbed dose
Abs_ing_particle	g/day	Ingestion: particle (indirect) absorbed dose
Abs_inh	g/day	Total absorbed inhalation dose
Abs_inh_after_use	g/day	Inhalation: far-field exposure (indirect) absorbed dose
Abs_inh_in_use	g/day	Inhalation: near-field exposure (direct) absorbed dose
Amt_inh_particle	g/day	Amount of chemical inhaled from particulate dust
Abs_dermal	g/day	Amount of chemical absorbed dermally
Abs_inh	g/day	Absorbed dose from all inhalation pathways (during use, after use, and indirect via particulate dust)
Abs_ing	g/day	Absorbed dose from all ingestion pathways (indirect incidental, indirect hand-to-mouth, indirect from surface particle resuspension)
Abs_Overall	g/day	Total absorbed dose across all pathways
ADME_rem	g/day	Total remaining internal body burden accounting for ADME processes after 1 day
Avail_oral	g	Mass of remaining chemical available on the skin after dermal absorption
C_in_use	g/m3	Adjusted airborne chemical concentration during product use set to a maximum of saturation concentration
C_in_use_calc	g/m3	Calculated airborne chemical concentration during product use
Chem_dust_frac	unitless	Mass fraction of dust comprising the chemical
Chem_floor	g	Mass of chemical on the floor after subtracting mass in air compartment
D	hours	Product use duration in hours (conversion of default use duration in minutes)
Dur_use_avg	minutes	Average product use duration in minutes for all products containing a specified chemical.
Dust_floor_mass	g	Total mass of dust on the floor of an indoor room
In_air	unitless	Fraction of chemical assigned to the air compartment based on Kaw
Intake_ing_dermal	g/day	Intake due to ingestion of chemical on skin from hand to mouth
Intake_ing_floor	g/day	Intake due to ingestion of chemical in floor dust from hand to mouth
Intake_ing_particle	g/day	Intake due to inhalation and subsequent ingestion of resuspended particles
Intake_ing_total	g/day	Total ingestion intake across all pathways
Intake_inh_after_use	g/day	Intake due to far-field inhalation exposure
Intake_inh_in_use	g/day	Intake due to near-field inhalation exposure
Intake_inh_particle	g/day	Intake due to inhalation of near-field direct particles
Intake_Inh_total	g/day	Total intake due to inhalation
JSS	g/(m2*day)	Transdermal flux
Mass_chem	g/day	Mass of chemical in amount of product used

Variable	Unit	Description
Mass_chem-air	g/day	Mass of chemical that goes into the air during product use
Mass_prod	g/day	Average mass of product used per day
Skin_load	g/day	Amount of chemical available on the skin for hand-to-mouth transfer
Sum_chem_cat_dermal	g/day	Weighted sum of chemical masses across product categories deposited on the skin (using product-category weights)
Sum_chem_cat_floor	g/day	Weighted sum of chemical masses across product categories deposited on the floor (using product-category weights)
Sum_chem_cat_inh	g/day	Weighted sum of chemical masses across product categories available for inhalation (using product-category weights and applying any outdoor dilution factors)
Sum_prod_cat	g/day	Total mass of products used per day across product categories after applying product-category-specific weights
Use_day	g/day	Average mass of product used per day
Use_day, air	g/day	Average effective mass of product per day that goes into the air, accounting for any outdoor dilution factor
Use_day, dermal	g/day	Average mass of product per day that goes onto the skin
Use_day, floor	g/day	Average mass of product per day that goes onto the floor

S3. Two-zone Model

Ex Priori applies the two-zone model of inhalation exposure, which is described thoroughly by Nicas [6]. Here, a derivation is presented for the scenarios modeled in Ex Priori: inhalation exposure during use and inhalation exposure after use.

S3.1. Inhalation Exposure during Use

Consider a room that has its air exchanged near the ceiling level. The air exchange rate is Q m^3/hour. Model this room as having two zones: a "room" zone with the volume V_{room}, and a "user bubble" zone with the volume V_{bubble} (the air space immediately surrounding a human product user). The air within each zone is perfectly mixed. Air is exchanged between the room and bubble zones at a rate of β m^3/hour.

Assume that the human opens a liquid product and starts to use it while still inside the user bubble. For example, they may be cleaning a surface using a disinfecting cleaner. This product contains a chemical of interest. The air concentration of the chemical of interest is initially zero, both in the user bubble and in the larger room. During product use, assume that the chemical is emitted from the product to the air at a constant rate *G* (g/hour). (We will discuss how to estimate *G* later.) The scenario is diagrammed in Figure S1.

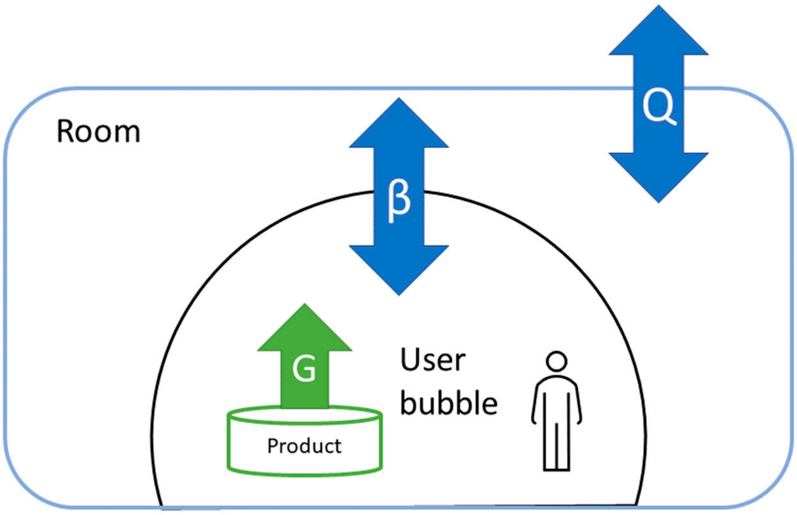

Figure S1. Diagram of two-zone model during product use showing the room zone and its air exchange rate with outside air; the user bubble zone and its air exchange rate with the room; the product and its emission rate; and the human receptor inside the user bubble.

The mass balance of the chemical of interest can then be described by a system of ODEs: Equation S55–Equation S57 [6]. C_{room} denotes the chemical concentration in the room air. C_{bubble} denotes the chemical concentration in user-bubble air.

Equation S55. Two-zone model ODEs: Cbubble

$$V_{bubble}\frac{dC_{bubble}}{dt} = -\beta C_{bubble}(t) + \beta C_{room}(t) + G(t) \quad \text{(S55)}$$

Equation S56. Two-zone model ODEs: Croom

$$V_{room}\frac{dC_{room}}{dt} = \beta C_{bubble}(t) - (Q + \beta)C_{room}(t) \quad \text{(S56)}$$

Equation S57. Two-zone model ODEs: G

$$\frac{dG}{dt} = 0 \quad \text{(S57)}$$

Nicas [6] solves this two-zone model in Appendix I of that publication and provides the resulting equations for time-dependent air concentration (g/m^3), which are reproduced here (Equation S58). (The originally published equations use C_{L} in place of C_{bubble} and C_{U} in place of C_{room}.)

Equation S58. Two-zone model solution during use: Cbubble

$$C_{\mathrm{bubble}}(t) = \alpha_1 \exp(\lambda_1 t) + \alpha_2 \exp(\lambda_2 t) + \alpha_3 \quad \text{(S58)}$$

Equation S59. Two-zone model solution during use: Croom

$$C_{\mathrm{room}}(t) = \alpha_1\left(\frac{\lambda_1 + a}{a}\right)\exp(\lambda_1 t) + \alpha_2\left(\frac{\lambda_2 + a}{a}\right)\exp(\lambda_2 t) + \alpha_3\frac{c}{d} \quad \text{(S59)}$$

Equation S60. Two-zone model solution during use: G

$$G(t) = \alpha_3\frac{ad - ac}{bd} \quad \text{(S60)}$$

The parameters λ_1, λ_2, a, b, c, and d are combinations of the basic model parameters β, Q, V_{bubble}, and V_{room} (Equation S61).

Equation S61. Parameters for two-zone model (lambdas and a, b, c, and d)

$$\lambda_1 = \frac{-(a+d) + \sqrt{(a+d)^2 - 4(ad - ac)}}{2}$$
$$\lambda_2 = \frac{-(a+d) - \sqrt{(a+d)^2 - 4(ad - ac)}}{2}$$
$$a = \beta / V_{\text{bubble}}$$
$$b = 1/V_{\text{bubble}}$$
$$c = \beta / V_{\text{room}}$$
$$d = (Q + \beta)/V_{\text{room}} \tag{S61}$$

Note that λ_1, λ_2, a, b, c, and d are all chemical-independent and product-independent. They do not depend on the chemical emission rate G or the duration of use of the product. Therefore, they are calculated only once in the "Scenario Inputs" tab of the Ex Priori model spreadsheet.

The coefficients α_1, α_2, and α_3 in Equation S58–Equation S60 can be found given the initial conditions described above: the air concentration of the chemical of interest is initially zero, both in the user bubble and in the larger room, and the emission rate G is constant. These initial conditions can be formulated as $C_{\text{bubble}}(0) = 0$, $C_{\text{room}}(0) = 0$, and $G(0) = G$. Nicas [6] shows that with some algebraic manipulation (see Appendix I of that publication), expressions can be obtained for α_1, α_2, and α_3 in terms of G, β, Q, V_{bubble}, λ_1, and λ_2 (Equation S62).

Equation S62. Alphas for two-zone model solution

$$\alpha_1 = G\left(\frac{\beta Q + \lambda_2 V_{\text{bubble}}(\beta + Q)}{\beta Q V_{\text{bubble}}(\lambda_1 - \lambda_2)}\right)$$
$$\alpha_2 = -G\left(\frac{\beta Q + \lambda_1 V_{\text{bubble}}(\beta + Q)}{\beta Q V_{\text{bubble}}(\lambda_1 - \lambda_2)}\right) \tag{S62}$$
$$\alpha_3 = \frac{G}{\left(\frac{\beta}{\beta + Q}\right) Q}$$

(Note that $\alpha_2 \neq -\alpha_1$: λ_2 appears in the numerator of α_1, but λ_1 appears in the numerator of α_2.)

S3.1.1. Cumulative inhalation exposure during product use

The cumulative inhalation exposure during product use is obtained by integrating the time-dependent inhalation exposure over the duration of product use. The time-dependent inhalation exposure during product use, $\text{Amt\_inh\_during}(t)$, is obtained by multiplying the inhalation rate (m^3/hour) by the time-dependent air concentration in the user bubble (g/m^3) (Equation S63). (Because Ex Priori defines the inhalation rate in units of m^3/day, it is divided by 24 to convert to m^3/hour.)

Equation S63. Amt_inh_during

$$\text{Amt\_inh\_during}(t) \;\; = \text{Inh\_rate}/24 \times C_{\text{bubble}}(t) \tag{S63}$$

The time-dependent air concentration in the user bubble, $C_{\text{bubble}}(t)$, was provided in Equation S58. Combining Equation S63 and Equation S58, we can write an expression describing $\text{Amt\_inh\_during}(t)$ (Equation S64).

Equation S64. Time-dependent amount inhaled during product use

$$\text{Amt\_inh\_during}(t) = \; = \text{Inh\_rate}/24 \times (\, \alpha_1 \exp\,(\lambda_1 t) + \alpha_2 \exp\,(\lambda_2 t) + \alpha_3) \tag{S64}$$

Now, in order to calculate the cumulative inhalation exposure during product use, we need to integrate Equation S64 over the duration of product use. Happily, we can separate out terms in the sum. Note that Ex Priori defines the duration of product use in minutes; we will divide by 60 to convert this to hours. For compactness, define $D \equiv \text{Dur\_use}/60$. The integral is written in Equation S65.

Equation S65. Integrating amount inhaled from user bubble over use duration

$$\int_0^D \text{Amt\_inh\_during}(t)\,dt = \text{Inh\_rate}/24 \times \left(\alpha_1 \int_0^D \exp(\lambda_1 t)\,dt + \alpha_2 \int_0^D \exp(\lambda_2 t)\,dt + \alpha_3 \int_0^D dt \right) \tag{S65}$$

Using the fact that $\int \exp(at)\,dt = \frac{\exp(at)}{a}$, we can work out the integral and derive a final expression for the cumulative amount of chemical inhaled during product use (Equation S66).

Equation S66. Cumulative amount inhaled during product use

$$\text{Amt\_inh\_during\_product use} = \text{Inh\_rate}/24 \times \left\{ \frac{\alpha_1}{\lambda_1}[\exp(\lambda_1 D) - 1] + \frac{\alpha_2}{\lambda_2}[\exp(\lambda_2 D) - 1] + \alpha_3[D] \right\} \tag{S66}$$

S3.2. Inhalation Exposure after Product Use

Consider the scenario after using a product. At the very end of product use, the air concentration of the chemical of interest in the user bubble and in a larger room can be obtained via the time-dependent equations (Equation S58–Equation S59) evaluated at time $t = D$, where D is the duration of product use. Then, product use stops, and therefore, new emissions also stop: $G = 0$ (here, we neglect any ongoing emissions that may occur from product lingering on surfaces after use). Now, air concentrations will "decay", as contaminated air moves out of the user bubble and out of the room, and fresh air moves in. Nicas [6] solves the two-zone model for this scenario in Appendix II of that publication and provides the resulting equations for time-dependent air concentration (g/m^3), which are reproduced below with updated notation (Equation S67–Equation S68). Here, t_{decay} represents the elapsed time since the end of product use (the elapsed time during the concentration "decay" phase). In other words, $t_{\text{decay}} = t - D$, where t represents the elapsed time since the beginning of product use, and product use is of duration D.

Equation S67. Time-dependent air concentrations after product use: Cbubble

$$C_{bubble}(t_{\text{decay}}) = \delta_1 \exp(\lambda_1 t_{\text{decay}}) + \delta_2 \exp(\lambda_2 t_{\text{decay}}) \tag{S67}$$

Equation S68. Time-dependent air concentrations after product use: Croom

$$C_{room}(t_{\text{decay}}) = \delta_1 \left(\frac{\lambda_1 + a}{a}\right) \exp(\lambda_1 t_{\text{decay}}) + \delta_2 \left(\frac{\lambda_2 + a}{a}\right) \exp(\lambda_2 t_{\text{decay}}) \tag{S68}$$

The coefficients λ_1 and λ_2 as well as a are the same as previously defined in Equation S61. However, the coefficients δ_1 and δ_2 are new. Their values must be derived for a specified set of initial conditions.

Nicas [6] finds δ_1 and δ_2 for the initial condition in which a contaminant was emitted at a constant rate in the user bubble for a long enough time that both the bubble and room air concentrations reached steady state—that is, where the initial conditions for the "decay" equations (Equation S67–Equation S68) are provided by the *steady-state* solutions of the "growth" equations (Equation S58–Equation S59). However, this is not the scenario modeled by Ex Priori. The duration of product use is unlikely to be long enough to achieve steady state. Therefore, we do not use the values of δ_1 and δ_2 provided by Nicas [6] but solve for them ourselves instead using the appropriate initial conditions for the scenario modeled by Ex Priori.

In the scenario modeled by Ex Priori, the initial conditions for the "decay" phase are simply the bubble and room air concentrations at the end of product use: that is, the initial conditions for the "decay" equations (Equation S67–Equation S68) at time $t_{\text{decay}} = 0$ are given by the "growth" equations (Equation S58–Equation S59) evaluated at time t = D (the product use duration in hours). These initial conditions are formulated in Equation S69 and Equation S70. The left-hand sides of Equation S69 and Equation S70 are obtained by setting $t_{\text{decay}} = 0$ in Equation S67 and Equation S68, respectively, and the right-hand sides are obtained by setting t = D in Equation S58 and Equation S59, respectively.

Equation S69. Initial condition in user bubble at end of product use

$$C_{\text{bubble}}\left(t_{\text{decay}} = 0\right) = \delta_1 + \delta_2 = \alpha_1 \exp\left(\lambda_1 \mathrm{D}\right) + \alpha_2 \exp\left(\lambda_2 \mathrm{D}\right) + \alpha_3 \tag{S69}$$

Equation S70. Initial condition in larger room at end of product use

$$C_{\text{room}}\left(t_{\text{decay}} = 0\right) = \delta_1\left(\frac{\lambda_1 + a}{a}\right) + \delta_2\left(\frac{\lambda_2 + a}{a}\right) = \alpha_1\left(\frac{\lambda_1 + a}{a}\right)\exp\left(\lambda_1 \mathrm{D}\right) + \alpha_2\left(\frac{\lambda_2 + a}{a}\right)\exp\left(\lambda_2 \mathrm{D}\right) + \alpha_3 \frac{c}{d} \tag{S70}$$

Now, to solve for δ_1 and δ_2, we need to use some algebra.
First, solve Equation S69 for δ_1.
Equation S71. Solving for delta1 in terms of delta2

$$\delta_1 = \alpha_1 \exp\left(\lambda_1 D\right) + \alpha_2 \exp\left(\lambda_2 D\right) + \alpha_3 - \delta_2 \tag{S71}$$

Substitute Equation S71 into Equation S70 and solve for δ_2.
Substituting Equation S71 into Equation S70:

$$\begin{gathered}\alpha_1\left(\frac{\lambda_1 + a}{a}\right)\exp\left(\lambda_1 D\right) + \alpha_2\left(\frac{\lambda_1 + a}{a}\right)\exp\left(\lambda_2 D\right) + \alpha_3\left(\frac{\lambda_1 + a}{a}\right) - \delta_2\left(\frac{\lambda_1 + a}{a}\right) + \delta_2\left(\frac{\lambda_2 + a}{a}\right) \\ = \alpha_1\left(\frac{\lambda_1 + a}{a}\right)\exp\left(\lambda_1 D\right) + \alpha_2\left(\frac{\lambda_2 + a}{a}\right)\exp\left(\lambda_2 D\right) + \alpha_3 \frac{c}{d}\end{gathered} \tag{S72}$$

The first term on either side, $\alpha_1\left(\frac{\lambda_1+a}{a}\right)\exp\left(\lambda_1 D\right)$, will be canceled (Equation S73).
Equation S73. Cancel first term from Equation S72:

$$\alpha_2\left(\frac{\lambda_1 + a}{a}\right)\exp\left(\lambda_2 D\right) + \alpha_3\left(\frac{\lambda_1 + a}{a}\right) + \delta_2\left(\frac{\lambda_2 - \lambda_1}{a}\right) = \alpha_2\left(\frac{\lambda_2 + a}{a}\right)\exp\left(\lambda_2 D\right) + \alpha_3 \frac{c}{d} \tag{S73}$$

Now rearrange Equation S73 to make sure that δ_2 is on one side by itself.
Equation S74. Rearrange Equation S73

$$\delta_2\left(\frac{\lambda_2 - \lambda_1}{a}\right) = \alpha_2\left(\frac{\lambda_2 + a}{a}\right)\exp\left(\lambda_2 D\right) + \alpha_3 \frac{c}{d} - \alpha_2\left(\frac{\lambda_1 + a}{a}\right)\exp\left(\lambda_2 D\right) - \alpha_3\left(\frac{\lambda_1 + a}{a}\right) \tag{S74}$$

Finally, divide through by $\left(\frac{\lambda_2-\lambda_1}{a}\right)$, or equivalently, multiply through by $\left(\frac{a}{\lambda_2-\lambda_1}\right)$ to yield Equation S75.

Equation S75. Solution of Equation S74 for delta2

$$\delta_2 = \alpha_2\left(\frac{\lambda_2 + a}{\lambda_2 - \lambda_1}\right)\exp\left(\lambda_2 D\right) + \alpha_3 \frac{c}{d}\left(\frac{a}{\lambda_2 - \lambda_1}\right) - \alpha_2\left(\frac{\lambda_1 + a}{\lambda_2 - \lambda_1}\right)\exp\left(\lambda_2 D\right) - \alpha_3\left(\frac{\lambda_1 + a}{\lambda_2 - \lambda_1}\right) \tag{S75}$$

Simplifying, Equation S75 becomes Equation S76.
Equation S76. Final expression for delta2

$$\delta_2 = \alpha_2 \exp\left(\lambda_2 D\right) + \left(\frac{\alpha_3}{\lambda_2 - \lambda_1}\right)\left[\frac{a(c-d)}{d} - \lambda_1\right] \tag{S76}$$

Equation S76 provides us with an expression for δ_2 under the non-steady-state initial conditions we want to model. Then, to get an expression for δ_1, we substitute back into Equation S71:

Equation S77. Substitute Equation S76 back into Equation S71

$$\delta_1 = \alpha_1 \exp\left(\lambda_1 D\right) + \alpha_2 \exp\left(\lambda_2 D\right) + \alpha_3 - \alpha_2 \exp\left(\lambda_2 D\right) - \left(\frac{\alpha_3}{\lambda_2 - \lambda_1}\right)\left[\frac{a(c-d)}{d} - \lambda_1\right] \tag{S77}$$

Simplifying, this becomes:
Equation S78. Final expression for delta1

$$\delta_1 = \alpha_1 \exp\left(\lambda_1 D\right) + \alpha_3\left[1 - \left(\frac{1}{\lambda_2 - \lambda_1}\right)\left(\frac{a(c-d)}{d} - \lambda_1\right)\right] \tag{S78}$$

Finally, this allows us to model the time-dependent air concentrations after product use is stopped. We take the equations for the time-dependent concentrations in the user bubble and room (Equation S67 and Equation S68, respectively) and substitute in δ_2 (defined in Equation S76); δ_1 (defined in Equation S78); and λ_1, λ_2, and a (defined in Equation S61).

Note that δ_1 and δ_2 are chemical-specific because they depend on α_1, α_2, and α_3, which, in turn, depend on the constant emission rate G that occurs during product use,

which, in turn, is determined by the chemical-specific quantities K_{aw}, Mass_chem, Mass_prod, and duration of product use D (see Equation S9 and discussion in S3.3). δ_1 and δ_2 also explicitly depend on D (in Equation S76 and Equation S78).

S3.2.1. Cumulative Inhalation Exposure after Product Use

We can calculate the cumulative inhalation exposure after product use, again, by integrating $C_{bubble}(t)$ as defined in Equation S67 over time for the rest of the day after product use has stopped (Equation S79). (Counting elapsed time from the end of product use to the end of the day, the lower limit of integration is 0, and the upper limit of integration is 24 hours minus the duration of product use.)

Equation S79. Integrating amount inhaled for the rest of the day after product use

$$\int_0^{24-D} \text{Amt\_inh\_after}(t_{\text{decay}})\, dt_{\text{decay}} = \text{Inh\_rate}/24 \times \left(\delta_1 \int_0^{24-D} \exp\left(\lambda_1 t_{\text{decay}}\right) dt_{\text{decay}} + \delta_2 \int_0^{24-D} \exp\left(\lambda_2 t_{\text{decay}}\right) dt_{\text{decay}} \right) \quad \text{(S79)}$$

This integral can be solved to obtain an analytical expression for the cumulative inhalation exposure that occurs after product use (Equation S80).

Equation S80. Cumulative amount inhaled for the rest of the day after product use

$$\text{Amt\_inh\_after} = \text{Inh\_rate}/24 \times \left\{ \frac{\delta_1}{\lambda_1}\left[\exp\left(\lambda_1(24-D)\right) - 1\right] + \frac{\delta_2}{\lambda_2}\left[\exp\left(\lambda_2(24-D)\right) - 1\right] \right\} \quad \text{(S80)}$$

S3.3. Estimating Emissions Rate

The cumulative amount of chemical inhaled during product use (Amt_inh_during_product use, Equation S66) and after product use (Amt_inh_after, Equation S80) depend on α_1, α_2, and α_3 (Equation S62), which, in turn, depend on G, the constant emissions rate during product use. Therefore, we still need to figure out a value for *G*, which will be both chemical-specific and product-specific.

S3.3.1. Conservative Approach: Assume all Chemical is Emitted

A common, conservative approach to estimating the emissions rate is to assume that *all* of the chemical in the consumer product will diffuse into the air during use [17]. Under this assumption, the constant emissions rate is given by Equation S81.

Equation S81. G, when all Mass_chem emitted

$$G = \text{Mass\_chem/Dur\_use} \quad \text{(S81)}$$

However, this approach makes no account of the differences in volatility between chemicals. For less-volatile chemicals, especially those with shorter average product use durations, it is unrealistic to expect all of the chemical in the product to be emitted into the air during product use. Therefore, Equation S81 is probably overly conservative for less-volatile chemicals. Furthermore, Equation S81 assumes that all available chemical mass is emitted into the air, leaving none for dermal or ingestion exposure pathways (i.e., the fraction In_air would always be 1).

S3.3.2. Most Detailed Approach: Time-dependent Emissions Models

To account for volatility, a time-dependent emission model would be the most correct. Time-dependent emission rates have been predicted in several published models of chemical volatilization based on chemical properties (such as vapor pressure, molecular weight, octanol–water partition coefficients). However, these are scenario-specific and are usually developed based on data gathered for only one or a few chemicals. Scenarios include spills of organic solvents [18]; pesticides applied to soils [19, 20]; quiescent pools of chemical [21, 22]; residential dishwashers [23]; residential washing machines [24-26]; and surface cleaning of residential countertops and floors [27, 28]. For example, the Consumer Exposure Model (CEM) [15] includes several different time-dependent emission scenarios

for different product types and use scenarios, including a product applied to a surface indoors; a sprayed product; a product added to water; or a product placed in the environment. The CEM solves a two- or three-zone air concentration model numerically using the appropriate time-dependent emission scenario.

One could even apply a time-dependent diffusion model, such as the one used by Earnest and Corsi [29], which is reproduced in Equation S82.

Equation S82. G: time-dependent diffusion

$$G(t) = k_g\left(HC_{chem-liquid}(t) - C_{chem-bubble}(t)\right) \quad \text{(S82)}$$

In that model, k_g is a mass-transfer coefficient (m/hr), H is the chemical's Henry's law coefficient, $C_{chem-liquid}$ is the chemical concentration in the liquid product, $C_{chem-bubble}$ is the time-varying chemical concentration in the user bubble, and A is the surface area of product application. The mass-transfer coefficient is chemical-specific but is not well defined; Earnest and Corsi [29] substitute a non-chemical-specific default value of 1 m/hr. The surface area of application will vary substantially depending on the product use scenario.

However, a time-dependent emission approach such as this one requires user specifications of product application type (to determine the correct time-dependent emissions model) as well as a numerical solution of the two-zone model rather than an analytical solution, as presented in S3.1 and S3.2. These data and computational requirements make such an approach infeasible for use with Ex Priori, which does not require detailed user input on the product application method, and is Excel-based such that numerical integration of the two-zone model is computationally difficult.

S3.3.3. Compromise Approach: Steady-state Scenario

To support Ex Priori's goal of rapid exposure-based chemical prioritization with minimal data requirements while avoiding the over-conservatism of assuming all chemical mass is emitted into the air, we developed a "compromise model" of a constant chemical emission rate based on a steady-state scenario. This steady-state scenario is not used directly to model inhalation exposure. Instead, it is used to estimate a constant emissions rate, which is then used with the time-dependent two-zone model solutions to model inhalation exposure.

The steady-state scenario in question is described as follows: If a volume of chemical-containing liquid product, V_{liquid}, is allowed to stand in contact with a volume of air, V_{air}, for enough time that the air and liquid chemical concentrations reach steady state, then the steady-state ratio of the air concentration to the liquid concentration is described by the chemical's air–water partitioning coefficient (K_{aw} from Henry's law). K_{aw} is usually known for a chemical a priori, either from experimental measurements or from QSAR-based model predictions.

How does this steady-state scenario apply to emissions in the two-zone model? Clearly, the air concentration inside the user bubble will not reach steady state during product use because air is constantly being exchanged. However, we reason as follows: Over the duration of use, the product is in contact with a *total* volume of air (V_{air}) equal to the total amount of air that flows through the user bubble in that time. We consider the hypothetical situation where the product stands in contact with that total volume of air *all at once*, with no air exchange, for long enough that the concentrations reach steady state. In that scenario, we can calculate the total amount of chemical that would be emitted. We then assume that the same total amount of chemical would be emitted during actual use, under non-steady state conditions. We further assume that that total amount of chemical is emitted at a constant rate during the duration of product use.

To find V_{air}, recall that in the two-zone model, air is exchanged between the room and user-bubble zones at a rate of β m^3/hour. The total volume of air V_{air} that flows through the user bubble during a product use duration D (hours) is provided by Equation S83.

Equation S83. V_air

$$V_{air} = \beta D \tag{S83}$$

If we placed the chemical-containing product in contact with that volume of air for a long-enough time for diffusion to reach steady-state, then the ratio of the air concentration and product concentration would be equal to K_{aw} (Equation S84).

Equation S84. Kaw definition: ratio of C_chem-air to C_chem-liq

$$K_{aw} = \frac{C_{chem-air}}{C_{chem-liq}} \tag{S84}$$

Under this steady-state assumption, the total mass of the chemical is equal to the mass in the liquid product plus the mass in the air (Equation S85).

Equation S85. Mass balance between air and liquid

$$Mass_{chem} = Mass_{chem-liq} + Mass_{chem-air} = C_{chem-liq} \times V_{liq} + C_{chem-air} \times V_{air} \tag{S85}$$

Then, we can solve Equation S85 for the concentration in the air, $C_{chem-air}$ (Equation S86). (Assume that the product is liquid with density ρ_{prod} (g/cm^3).)

Equation S86. Solve for C_chem-air

$$C_{chem-air} = \frac{K_{aw} \times Mass_{chem}}{\frac{Mass_{prod}}{\rho_{prod}} + K_{aw} \times V_{air}} \tag{S86}$$

Check to make sure that this predicted $C_{chem-air}$ does not exceed the saturation concentration of the chemical in air (C_{sat}), where C_{sat} is predicted using Equation S87, a QSAR model used in the Consumer Exposure Model [15]. VP is the chemical's vapor pressure in Pascals; MW is the chemical's molecular weight in g/mol; R is the gas constant; T is the temperature in Kelvin (room temperature assumed 298 K).

Equation S87. Csat QSAR

$$C_{sat} = \frac{\mathrm{VP} \times \mathrm{MW}}{RT} \tag{S87}$$

The adjusted air concentration is then given by the lesser of $C_{chem-air}$ and C_{sat}.

Equation S88. C_chem-air,adj

$$C_{chem-air,adj} = \min(C_{chem-air}, C_{sat}) \tag{S88}$$

The total mass of chemical in the air is then simply the air concentration ($C_{chem-air,adj}$ given by Equation S88) multiplied by the air volume (V_{air} given by Equation S83).

Equation S89. Mass_chem-air

$$Mass_{chem-air} = C_{chem-air,adj} \times V_{air} = \beta D \times \min\left(\frac{K_{aw} \times Mass_{chem}}{\frac{Mass_{prod}}{\rho_{prod}} + K_{aw} \times \beta D}, \frac{\mathrm{VP} \times \mathrm{MW}}{RT}\right) \tag{S89}$$

To estimate the emission rate G (g/hour), assume that $Mass_{chem-air}$ is emitted at a constant rate over the duration of product use.

Equation S90. G derived from Mass_chem-air

$$G = \frac{Mass_{chem-air}}{D} = \beta \times \min\left(\frac{K_{aw} \times Mass_{chem}}{\frac{Mass_{prod}}{\rho_{prod}} + K_{aw} \times \beta D}, \frac{\mathrm{VP} \times \mathrm{MW}}{RT}\right) \tag{S90}$$

In this way, the effects of time-dependent diffusion, occurring over potentially short product use durations, are roughly approximated by using a steady-state approximation with a duration-dependent value of $V_{air} = \beta D$.

S3.4. Sensitivity of Inhalation Exposure to β

For Ex Priori, we have adopted the value of β used by the Consumer Exposure Model [15] (found in Appendix B, Table B-14 of that publication), i.e., 82.008 m^3/hour or 1.3668 m^3/min. However, the parameter β lacks sufficient experimental data to determine its value. The little available experimental data estimate a wide possible range of values. Available data come from laboratory studies involving heated mannequins to attempt to model convection-driven air movement from a warm human body: these studies have estimated values for β of between 1 and 5 m^3/min [30]. However, it should be noted that these studies were based on air measurements of only one chemical: toluene. It is not clear whether there may be chemical-dependent error, bias, or uncertainty in the estimates of β. Other studies have estimated β from measurements of the random air speed near the boundary of the user bubble [18, 31]. In a room with a volume of 22.7 m^3 and an air supply rate of 18.6 m^3/min, β was estimated [18] at 2.7 m^3/min by assuming a hemispheric user bubble of radius 0.5 m and using a measured random air speed of 3.4 m/min. In an industrial occupational setting, β was estimated [31] to be between 7 and 11 m^3/min, although these numbers may not be particularly relevant to the residential consumer setting modeled by Ex Priori. For β, the "default" value suggested by Nicas [6] is β = 180 m^3/hour, or 3 m^3/min, based on assuming a hemispheric user bubble with a radius of 0.46 m and an average random air speed of 4.6 m/min.

For the qualitative sensitivity analysis presented in the main text, we selected a lower bound of β = 1 m^3/min and an upper bound of β = 5 m^3/min based on the analysis of Zhang, Banerjee [30]. This sensitivity analysis showed that Ex Priori-predicted median inhalation exposure increased at the upper-bound value of β. In this section, we further investigate the details of sensitivity of predicted inhalation exposures to β and elucidate some chemical-specific sensitivity effects.

Modeled inhalation exposure depends on β in two different and opposing ways. First, a larger value of β means that a larger volume of air passes through the user bubble during product use. Under the constant-emissions steady-state approximation described above, this allows more chemical mass to be emitted to the air, which tends to increase inhalation exposure. Second, a larger value of β allows the emitted chemical to be cleared more quickly from the user bubble, which tends to decrease inhalation exposure.

Which of these two opposing effects will govern? The answer depends on how much of the available chemical mass is emitted into the air (according to the constant-emissions steady-state approximation), even at the lower-bound value of β (1 m^3/min).

To illustrate, let us examine sensitivity to β for two chemicals: toluene (CASRN 108-88-3) and isopropyl myristate (CASRN 110-27-0). These two chemicals were chosen because Ex Priori ranks them highly under default conditions, and they have very different volatility levels (see following discussion). The analytical, time-dependent solution of the two-zone model was implemented in R, following Equations S54–S56 and Equation S60. The model was evaluated for each chemical for five different values of β: 1, 2, 3, 4, and 5 m^3/min. Parameters for each chemical are provided in Table S6.

Table S6. Parameters used for analytical, time-dependent inhalation model solution for two example chemicals: toluene and isopropyl myristate.

Parameter	Chemical	
	**Toluene**	**Isopropyl Myristate**
K_{aw}	0.243	3.09×10^{-5}
Dur_use, min (average across products)	50.5	8.8
Total product mass, g	123.3	136.6
Total chemical mass, g	17.2	8.5
Inhalation rate, m^3/day	16.2	
V_{bubble}, m^3	0.2	
V_{room}, m^3	5.8 × 5.8 × 2.4	

Q, m^3/minute	0.605

The results (Figure S2) are striking: these two chemicals exhibit very different sensitivities to β. For toluene, inhalation exposure decreases asymptotically as β increases. For isopropyl myristate, inhalation exposure increases linearly was β increases.

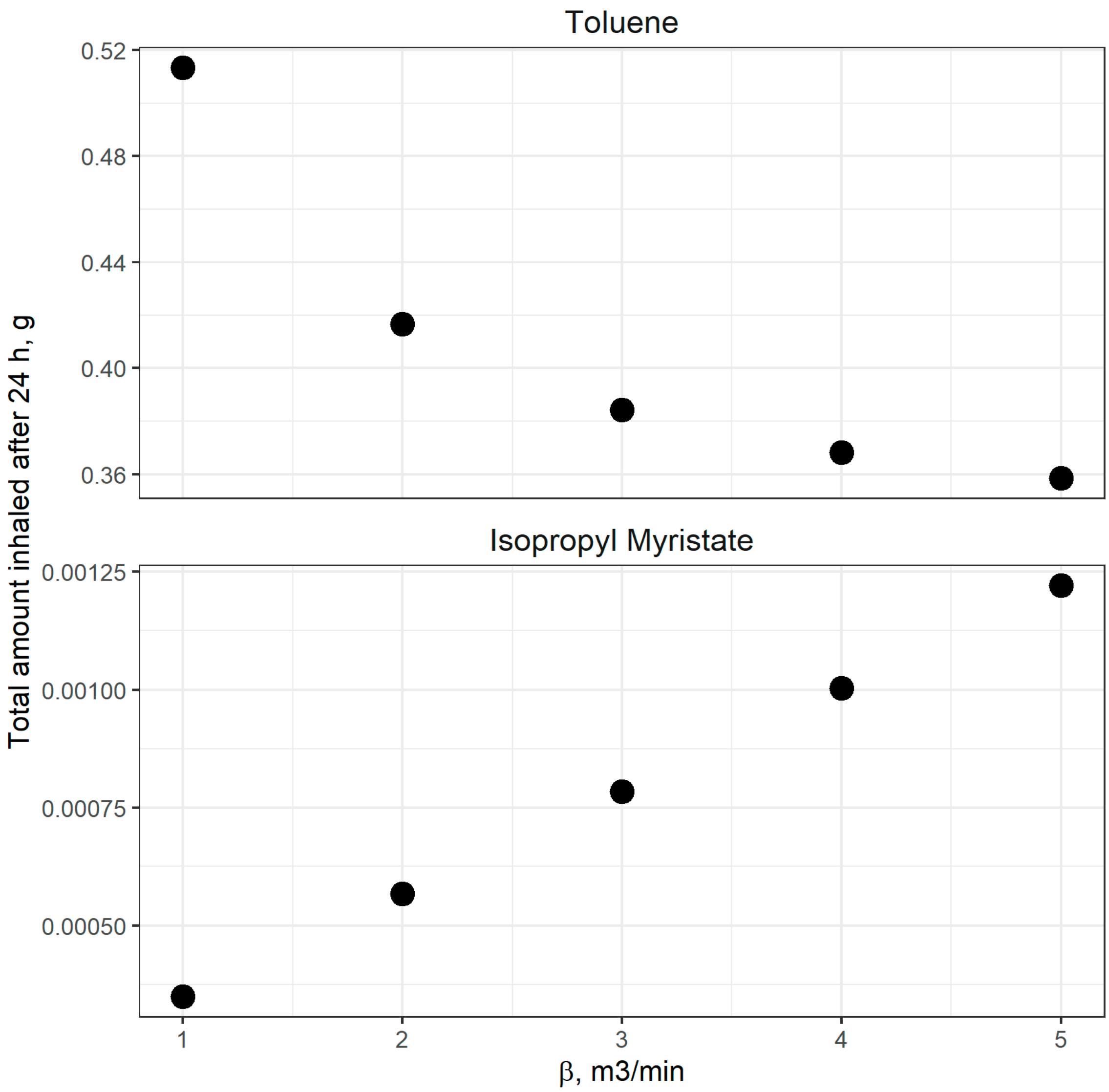

Figure S2. Points: Total amounts inhaled over 24 hours for toluene (upper panel) and isopropyl myristate (lower panel) for five different values of β.

The sources of the difference can be explored by examining the time course of the concentration in the user bubble (C_{bubble}) and how it changes for different β values (Figure S3). Only one hour of time is shown in Figure S3 to allow observations of the detailed kinetics during and just after product use. For toluene, the peak concentration (at the end of product use) decreases asymptotically as β increases, whereas for isopropyl myristate, the peak concentration increases approximately linearly as β increases.

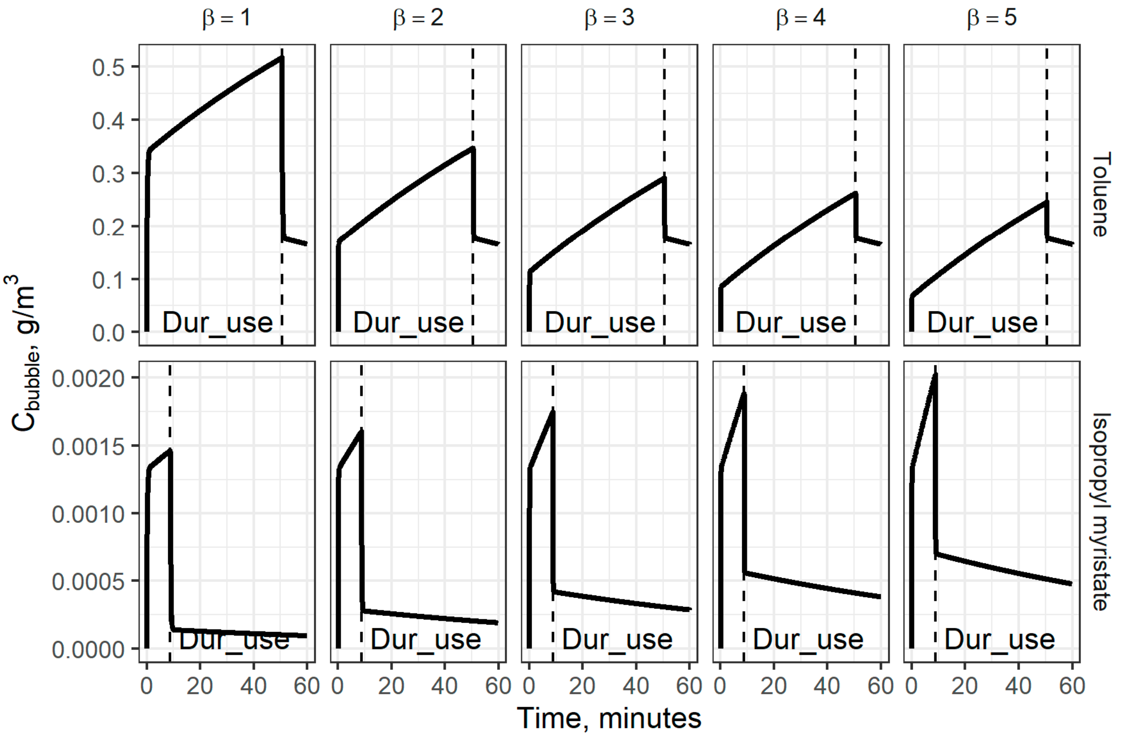

Figure S3. Inhalation model predicted for Cbubble vs. time (over the course of one hour elapsed after the beginning of product use) for five different β values (columns). Top row: Toluene. Bottom row: Isopropyl myristate. In each panel, the end of product use is marked by a vertical black line labeled "Dur_use" (representing the average product use duration for products containing each chemical: 50.5 minutes for toluene; 8.8 minutes for isopropyl myristate).

In turn, we asked what drives the changes in C_{bubble} as β increases. C_{bubble} is the balance between the amount of chemical entering the user bubble and the amount leaving it. During product use, air exchange at rate β results in a net loss of chemical from the user bubble (because $C_{room} \leq C_{bubble}$, so as bubble air is replaced with room air, the net effect is a loss of chemical). This means that during product use, the overall increase in concentration in the user bubble is driven by the balance between net loss due to air exchange and the amount emitted from the product. These quantities and their balance change with β. To visualize the balance between these two quantities, we plot their values at the end of product use as a function of β (Figure S4). For toluene, the amount emitted is not sensitive to β, but the amount removed from the user bubble increases asymptotically with β. For isopropyl myristate, the amount emitted and the net amount removed both increases approximately linearly with β, but the amount emitted increases with a very slightly steeper slope. The difference may not be visually apparent in the lower panel of Figure S4, but it results in s small increase in peak C_{bubble} with β that is apparent in the bottom row of Figure S3.

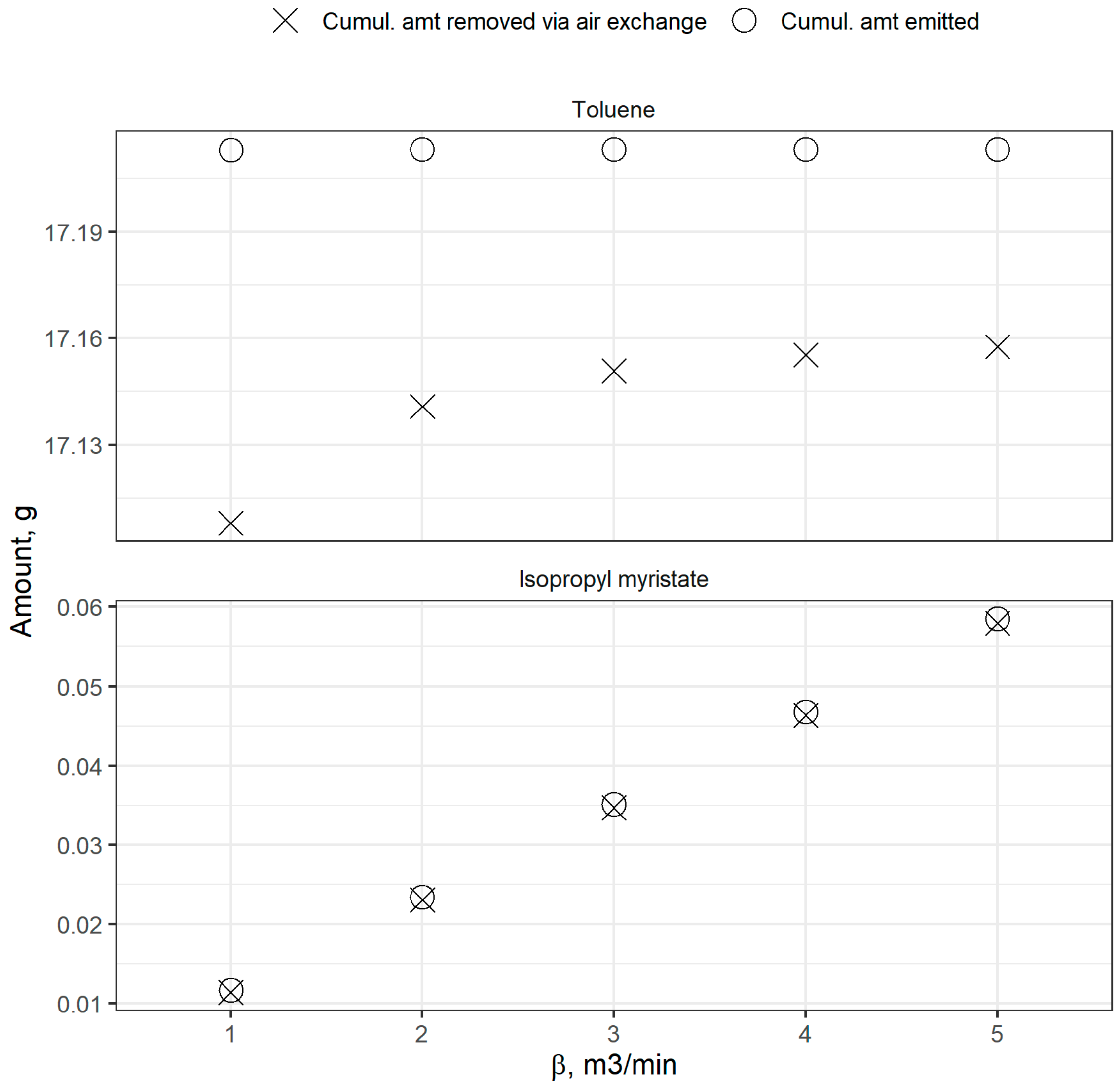

Figure S4. End of product use: Cumulative net chemical amount removed via air exchange from the bubble (X's) and cumulative chemical amount emitted (circles) for five different values of β.

In turn, what causes emissions to lack sensitivity to β for toluene but to increase with β for isopropyl myristate, as seen in Figure S4? The key is the assumptions of the steady-state scenario used to approximate constant emissions. The total amount of chemical emitted to the air ($Mass_{chem-air}$) is given by Equation S89: it depends on K_{aw}, $Mass_{chem}$, $Mass_{prod}$, Dur_use, and β. If this steady-state air concentration is greater than the air saturation concentration predicted by a QSAR (Equation S87), then $Mass_{chem-air}$ is taken to be the amount needed to achieve the saturation concentration.

In this scenario, increasing β effectively increases V_{air} and therefore increases the amount of chemical needed to reach the required steady-state or saturation air concentration in the larger volume of air. However, the form of Equation S89 indicates that $Mass_{chem-air}$ has an upper asymptote of $Mass_{chem}$. In fact, it can be rewritten by scaling variables as Equation S91:

Equation S91. "Scaled" version of Maschmeier

$$\frac{Mass_{chem-air}}{Mass_{chem}} = \min\left(non - saturated \; = \; \frac{x}{1+x}, saturated \; = \; Kx\right) \tag{S91}$$

where

Equation S92. Definition of x in scaled Mass_chem_air eqn

$$x \equiv \frac{K_{aw}\beta D}{\mathrm{Mass_{prod}}/\rho_{\mathrm{prod}}} \tag{S92}$$

and

Equation S93. Definition of K in scaled Mass_chem_air eqn

$$K \equiv \frac{VP \times MW \times \mathrm{Mass_{prod}}/\rho_{\mathrm{prod}}}{K_{aw} \times RT} \tag{S93}$$

$Mass_{chem-air}/Mass_{chem}$ approaches 1 as the quantity $K_{aw}\beta D/(\mathrm{Mass_{prod}}/\rho_{\mathrm{prod}})$ increases towards infinity (see Figure S5).

For the non-saturated case in Equation S91, the relation is illustrated as the curve in Figure S5 or as in Figure S6, where the x-axis is on a $\log_{10}$ scale. For the saturated case in Equation S91, the relation is a straight line with a chemical-specific slope K (Equation S93), so long as that line falls below the curve for the non-saturated case (gray lines in Figure S5; gray curves in Figure S6, where straight lines become curves because of the $\log_{10}$ scale for the x-axis). For a given β value, each chemical will have a chemical-specific x-value in Figure S5 or Figure S6 (given by Equation S92). To calculate $Mass_{chem-air}/Mass_{chem}$ for a given chemical and a given β value, first calculate the chemical-specific x-value $K_{aw}\beta D/(\mathrm{Mass_{prod}}/\rho_{\mathrm{prod}})$.

$Mass_{chem-air}$ drives the emissions rate G, which, in turn, drives concentration in the user bubble and thus drives total inhalation exposure both during and after product use. Therefore, the sensitivity of inhalation exposure to β depends on the steepness of the $Mass_{chem-air}$ relation in Equation S91 in the range of β under consideration (1-5 m^3/min). In other words, where does the chemical fall along the curve in Figure S5 or Figure S6 for β = 1-5 m^3/min: along the steep part or the shallow part approaching the asymptote? Additionally, if it falls on its chemical-specific saturated line in the range of β = 1-5 m^3/min, how steep is the slope of that line? If $Mass_{chem-air}$ is already approaching 100% of $Mass_{chem}$ at default β, then increasing β will result in only a very small increase in $Mass_{chem-air}$.

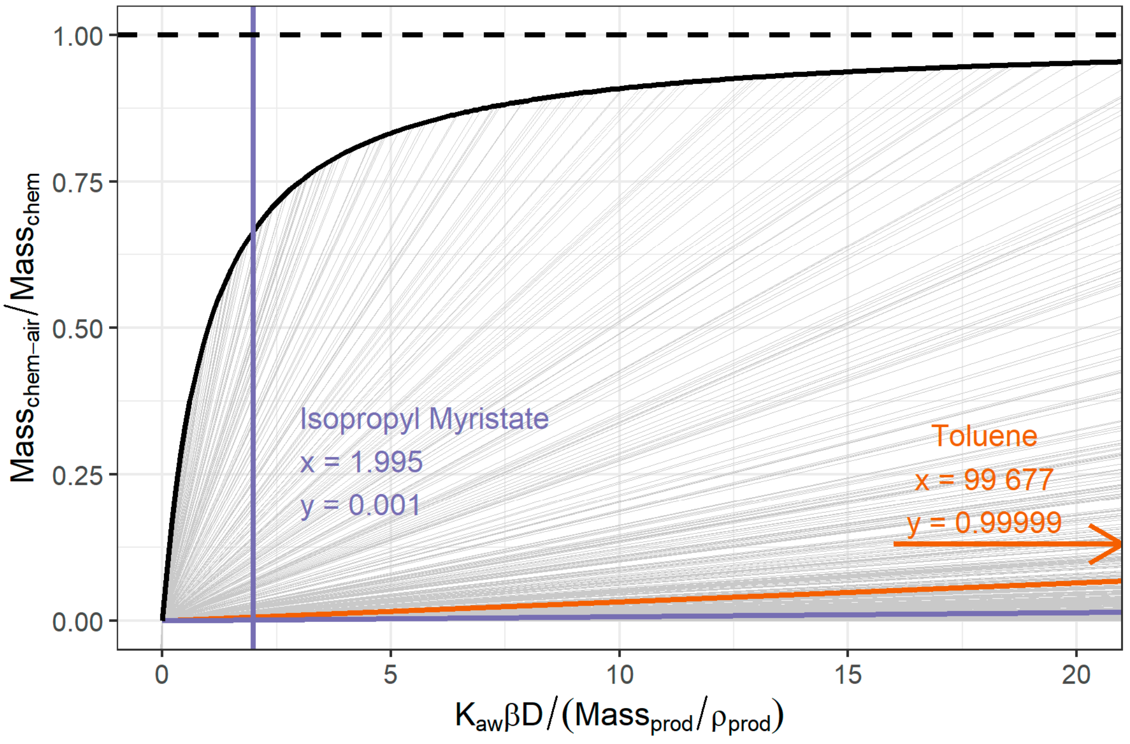

Figure S5. Black curve: General shape of the relation for the non-saturated case between $Mass_{chem-air}/Mass_{chem}$ and $K_{aw}\beta D/(Mass_{prod}/\rho_{prod})$. Gray lines: Chemical-specific relations for the saturated case. Dashed line: Upper asymptote indicating that $Mass_{chem-air}$ cannot be more than 100% of $Mass_{chem}$. Purple diagonal line: The saturated relation for isopropyl myristate. Purple vertical line: The x-axis value for isopropyl myristate at β = 1m³/min (here, 1.995). Where this vertical line crosses the saturated line and the non-saturated curve, we take the lower of the two y-values (here, 0.001, from the saturated line). Orange diagonal line: The saturated relation for toluene. Orange arrow indicates that the x-axis value for toluene is far beyond the limits of this plot (here, it is 99,677).

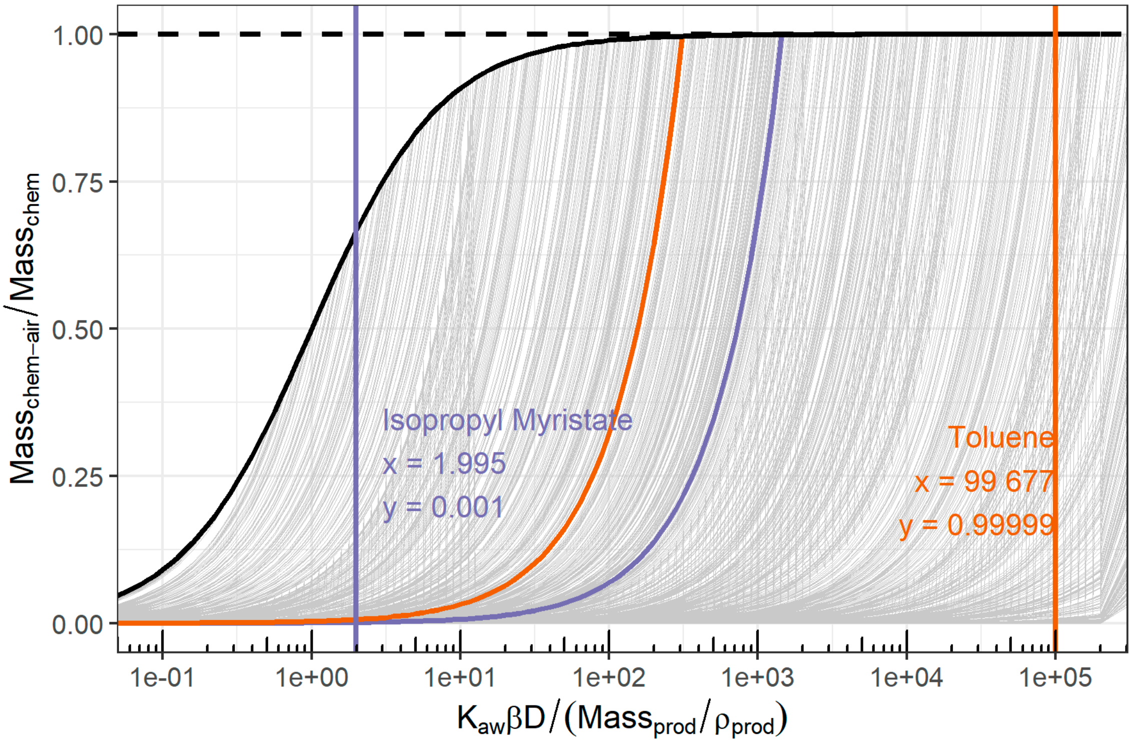

Figure S6. The same as Figure S5, but with a $\log_{10}$ scale for the x-axis and showing a x-axis range up to x = 150,000 so that toluene and isopropyl myristate can be seen on the same plot. On this $\log_{10}$ scale, the chemical-specific gray lines representing the saturated relations become gray curves; isopropyl myristate is highlighted in purple, and toluene in orange. Note that each saturation curve is drawn to end where it crosses the non-saturated black curve, highlighting the fact that the saturated value will only be used if it is less than the non-saturated value. Orange vertical line: The x-axis value for toluene at default β = 82.008/60 m³/min. This vertical line is well beyond the point where the orange saturated curve crosses the non-saturated black curve, so the y-axis is given by the non-saturated curve (here, 0.99999).

Toluene is on the extremely shallow part of this asymptotic relation, even for low-end β = 1 m³/min, as seen in Figure S6 and Figure S5. Increasing β therefore barely increases emissions at all. However, increasing β does increase the rate at which a chemical is removed from the user bubble via air exchange. On balance, then, increasing β tends to decrease yjr concentration in the user bubble.

Isopropyl myristate is limited by its air saturation concentration in the range β = 1-5 m³/min, meaning that sensitivity is defined by the chemical-specific slope of its saturation line (Equation S93). For this chemical K = 6.9e-4. Although this is a shallow slope that results in only a small increase in $Mass_{chem-air}$ as β increases from 1 to 5 m³/min, this increase still slightly outweighs the corresponding increase in the rate at which a chemical is removed from the user bubble via air exchange. On balance, then, increasing β tends to increase the concentration in the user bubble.

These results help to elucidate the results of the overall sensitivity analysis of the effect of β on inhalation exposure across all chemicals in the Ex Priori model, as presented

in Figure 3 of the main text. There, the low-end value of β was 60 m^3/hour (1 m^3/min), and the high-end value of β was 300 m^3/hour (5 m^3/min) compared to the default value of 82.008 m^3/hour (1.3668 m^3/min). In that sensitivity analysis, median inhalation exposure across chemicals increased at the high-end value of β. This result suggests that the median chemical in Ex Priori behaves more like isopropyl myristate than toluene.

This result can be elucidated further by calculating the ratio of inhalation exposure for high-end β to inhalation exposure for the default β for all of the chemicals in Ex Priori. If this ratio is greater than 1, then inhalation exposure increases at the high-end value of β; if the ratio is less than 1, then inhalation exposure decreases at the high-end value of β. This ratio can then be plotted vs. the fraction emitted, $Mass_{chem-air}/Mass_{chem}$, calculated at the default β = 82.008/60 = 1.4 m^3/min. Note that toluene and isopropyl myristate are both marked on Figure S7.

As shown in Figure S7, there are two "branches" of the relationship between the ratio and fraction emitted. One branch applies to chemicals that do not reach the air saturation concentration at default β; the other branch applies to chemicals that do reach saturation. The non-saturated branch corresponds to using the black curve in Figure S5, and the saturated branch corresponds to using the chemical-specific gray curve in Figure S5. The horizontally flat part of the saturated branch (where isopropyl myristate is marked) corresponds to the shallowest gray lines in Figure S5—those where increased emissions barely outpace increased air-exchange removal with increased β. The two points between the branches represent chemicals that reach air saturation concentration at default β but that are no longer saturated at high-end β. These are Sodium Octane-1-Sulphonate Monohydrate (CASRN 5324-84-5) and 2-Methyl-P-Phenylenediamine Sulphate (CASRN 6369-59-1). The five points on the saturated branch that are below the ratio-1 horizontal line (meaning that inhalation exposure decreased at high-end β for these five chemicals) were also saturated at default β but were no longer saturated at high-end β, meaning that the effective steepness of the dependence on β is reduced. Estimating according to Figure S7, the ratio of intake exposure for high to default β is approximately one when the fraction emitted is approximately 0.7.

As shown in the horizontal marginal histogram on Figure S7, the majority of chemicals considered in Ex Priori have been emitted at low fractions at default β, meaning that as β increases, they have a steep enough increase in emissions to outweigh increased removal via air exchange. Additionally, 56% of Ex Priori chemicals reach the air saturation concentration at default β and are therefore governed by the "saturated" branch of the relation. Compared to the non-saturated branch, chemicals on the saturated branch show a greater increase in inhalation exposure as β increases. This leads to the result observed in the main sensitivity analysis: the 50th percentile inhalation exposure increases when β is increased to its high-end value of 5 m^3/minute.

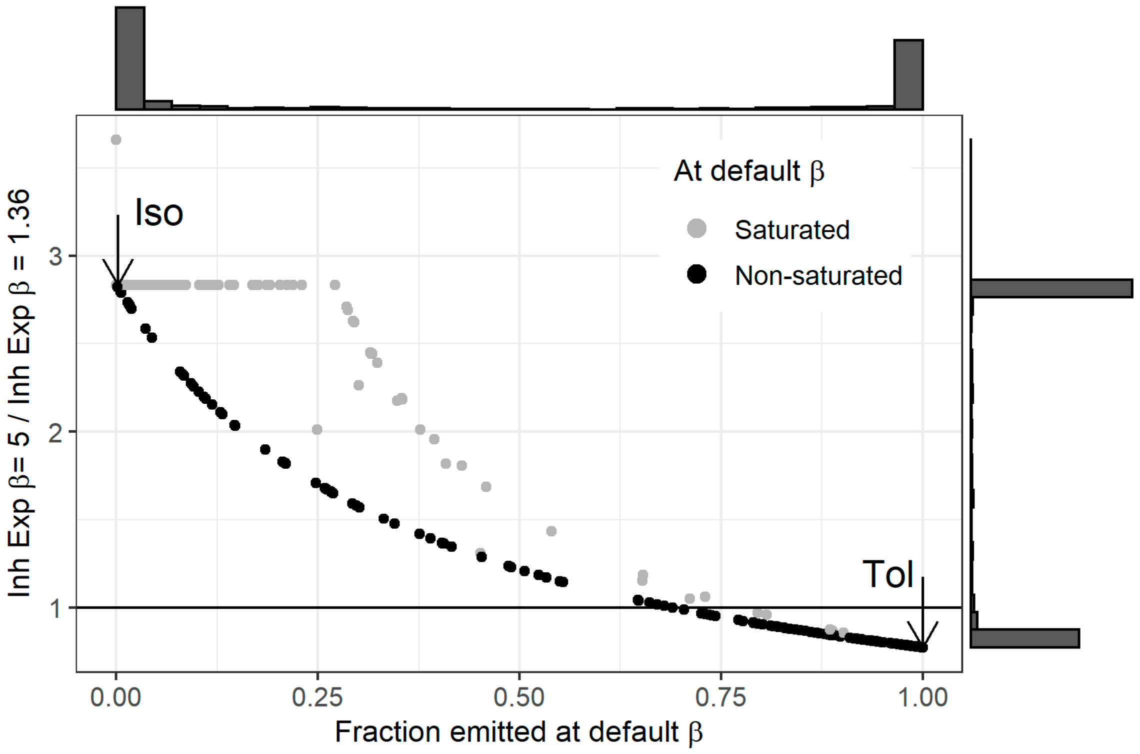

Figure S7. Points: Ratio of Ex Priori-predicted total inhalation exposure for high-end β (5 m³/min) to total inhalation exposure for default β (1.36 m³/min) plotted against fraction of chemicals emitted at default β. Each point is one chemical. Points are color-coded by whether the steady-state air concentration is saturated at default β (1.36 m³/min). Isopropyl myristate is marked with an arrow labeled "Iso". Toluene is marked with an arrow labeled "Tol." Horizontal line: An inhalation-exposure ratio of 1. Below this line, inhalation exposure decreases between default and high-end β. Above this line, inhalation exposure increases between default and high-end β. Margins: Histograms of fraction emitted and the ratio of inhalation exposures for high-end vs. default β (height of bars = count of chemicals in each bin).

Additionally, the sensitivity of the fraction emitted to β helps to explain the sensitivity of non-inhalation exposures to β. Because Ex Priori calculates chemical emissions to air *first* and divides the *remaining* chemical mass between skin and floor dust, the mass of chemical available for dermal or indirect ingestion exposure therefore depends on the mass of chemical emitted to air. The mass emitted to air is sensitive to β for all but the most volatile chemicals. Therefore, dermal and indirect ingestion exposures are indirectly sensitive to β as well for all but the most volatile chemicals.

S4. Evaluation of Ex Priori Predictions by Comparison to Population Aggregate Exposures Inferred from NHANES Urinary Biomonitoring Data

Table S7. Letter codes used in Figure 4 in main text to represent chemicals. Column 4 is the NHANES-inferred population median aggregate exposure drawn from Supplemental Table S4 of Stanfield et al. [32], and Column 5 is the Ex Priori-predicted absorbed exposure from the "Dashboard" tab of Ex Priori (run with all weights set equal to 1) converted from g/day to mg/kg/day by dividing by an average adult body weight of 70 kg and applying a conversion factor of 1000 mg/g.

Code	CAS.RN	Name	NHANES-inferred Median Aggregate Exposure (mg/kg/day)	Ex Priori-Predicted Absorbed Exposure (mg/kg/day)
**A**	108-88-3	Toluene	3.92×10^{-5}	6.29
**B**	131-57-7	Benzophenone-3	3.17×10^{-4}	6.07

Code	CAS.RN	Name	NHANES-inferred Median Aggregate Exposure (mg/kg/day)	Ex Priori-Predicted Absorbed Exposure (mg/kg/day)
**C**	94-26-8	Butyl paraben	$7.98{\times}10^{-7}$	3.27
**D**	1330-20-7	Xylene	$2.35{\times}10^{-3}$	2.47
**E**	106-46-7	para-Dichlorobenzene	$3.86{\times}10^{-5}$	2.23
**F**	120-47-8	Ethyl paraben	$1.56{\times}10^{-5}$	2.70
**G**	127-18-4	Tetrachloroethene	$1.94{\times}10^{-7}$	1.06
**H**	100-41-4	Ethylbenzene	$1.09{\times}10^{-4}$	1.06
**I**	94-13-3	n-propyl paraben	$7.34{\times}10^{-5}$	1.34
**J**	99-76-3	Methyl paraben	$5.54{\times}10^{-4}$	1.62
**K**	84-66-2	Diethyl phthalate	$6.33{\times}10^{-4}$	$5.59{\times}10^{-1}$
**L**	79-01-6	Trichloroethene	$4.35{\times}10^{-7}$	$3.89{\times}10^{-1}$
**M**	3380-34-5	Triclosan	$5.94{\times}10^{-5}$	$1.17{\times}10^{-1}$
**N**	84-74-2	Dibutyl phthalate	$1.33{\times}10^{-5}$	$9.00{\times}10^{-2}$
**O**	91-20-3	Naphthalene	$8.60{\times}10^{-6}$	$9.64{\times}10^{-2}$
**P**	101-20-2	Triclocarban	$1.04{\times}10^{-6}$	$5.49{\times}10^{-2}$
**Q**	121-75-5	Malathion	$9.01{\times}10^{-7}$	$7.33{\times}10^{-2}$
**R**	90-43-7	ortho-Phenylphenol	$8.13{\times}10^{-7}$	$2.38{\times}10^{-2}$
**S**	100-42-5	Styrene	$5.18{\times}10^{-4}$	$1.90{\times}10^{-2}$
**T**	85-68-7	Benzylbutyl phthalate	$1.96{\times}10^{-4}$	$1.21{\times}10^{-2}$
**U**	13674-84-5	Tris(1-chloro-2-propyl) phosphate (TCPP)	$3.05{\times}10^{-6}$	$1.75{\times}10^{-2}$
**V**	94-75-7	2,4-Dichlorophenoxyacetic acid	$4.18{\times}10^{-7}$	$2.12{\times}10^{-2}$
**W**	1912-24-9	Atrazine	$8.14{\times}10^{-8}$	$1.67{\times}10^{-2}$
**X**	134-62-3	N,N-Diethyl-3-methylbenzamide	$4.36{\times}10^{-7}$	$2.61{\times}10^{-2}$
**Y**	541-73-1	1,3-dichlorobenzene	$2.84{\times}10^{-7}$	$1.18{\times}10^{-2}$
**Z**	63-25-2	Carbaryl	$8.54{\times}10^{-6}$	$1.68{\times}10^{-2}$
**a**	52645-53-1	Permethrin	$1.19{\times}10^{-6}$	$4.94{\times}10^{-3}$
**b**	52315-07-8	Cypermethrin	$1.24{\times}10^{-6}$	$2.84{\times}10^{-3}$
**c**	52918-63-5	Deltamethrin	$1.38{\times}10^{-5}$	$1.93{\times}10^{-3}$
**d**	2921-88-2	Chlorpyrifos	$1.51{\times}10^{-6}$	$1.20{\times}10^{-3}$
**e**	126-73-8	Tri-n-butyl phosphate (TBuP)	$3.51{\times}10^{-6}$	$1.37{\times}10^{-3}$
**f**	71-43-2	Benzene	$2.84{\times}10^{-6}$	$1.69{\times}10^{-3}$
**g**	62-73-7	Dichlorvos	$9.22{\times}10^{-7}$	$5.38{\times}10^{-3}$
**h**	68359-37-5	Cyfluthrin	$5.55{\times}10^{-7}$	$4.36{\times}10^{-4}$
**i**	114-26-1	Propoxur	$3.06{\times}10^{-9}$	$1.45{\times}10^{-3}$
**j**	22248-79-9	Tetrachlorvinphos	$1.44{\times}10^{-6}$	$5.68{\times}10^{-4}$
**k**	75-56-9	Propylene oxide	$1.49{\times}10^{-4}$	$7.62{\times}10^{-2}$
**l**	52-68-6	Trichlorfon	$1.05{\times}10^{-6}$	$2.02{\times}10^{-3}$
**m**	123-30-8	4-Aminophenol	$1.22{\times}10^{-7}$	$7.06{\times}10^{-4}$
**n**	30560-19-1	Acephate	$4.39{\times}10^{-7}$	$2.84{\times}10^{-2}$
**o**	107-13-1	Acrylonitrile	$1.12{\times}10^{-5}$	$1.88{\times}10^{-4}$
**p**	79-06-1	Acrylamide	$3.85{\times}10^{-5}$	$1.94{\times}10^{-5}$

Here is the R summary of the linear regression model shown in Figure 4 of the main text (linear model of the data in Table S7), where "Median_Exposure" is the NHANES-inferred population median aggregate exposure drawn from Supplemental Table S4 of Stanfield et al. [32], and "ExPriori_abs_mgkgday" is the Ex Priori-predicted absorbed exposure from the "Dashboard" tab of Ex Priori (run with all weights set equal to 1)

converted from g/day to mg/kg/day by dividing by an average adult body weight of 70 kg and applying a conversion factor of 1000 mg/g.

```
Call:
lm(formula = log10(Median_Exposure) ~ log10(ExPriori_abs_mgkgday),
    data = ep_nhanes_adult)

Residuals:
     Min       1Q   Median       3Q      Max
-2.70614 -0.86411  0.01139  0.82316  2.11469

Coefficients:
                            Estimate Std. Error t value Pr(>|t|)
(Intercept)                  -4.7172     0.2667 -17.689  < 2e-16
log10(ExPriori_abs_mgkgday)   0.3845     0.1320   2.912  0.00584

(Intercept)                 ***
log10(ExPriori_abs_mgkgday) **
---
Signif. codes:  0 '***' 0.001 '**' 0.01 '*' 0.05 '.' 0.1 ' ' 1

Residual standard error: 1.159 on 40 degrees of freedom
Multiple R-squared: 0.1749, Adjusted R-squared: 0.1543
F-statistic: 8.482 on 1 and 40 DF; p-value: 0.005843
```

Here is the R summary of the unweighted linear regression between the SEEM3-predicted median population aggregate exposures and the NHANES-inferred median aggregate exposures (using the most recent NHANES cohort). Data are drawn directly from Supplemental Table S9 from Stanfield et al. [32]:

```
Call:
lm(formula = log10(Bayesmarker_median) ~ log10(SEEM_median),
    data = s9_recent)

Residuals:
    Min      1Q  Median      3Q     Max
-3.3562 -1.5204  0.3306  1.5718  3.2528

Coefficients:
                   Estimate Std. Error t value Pr(>|t|)
(Intercept)          0.7463     2.3118   0.323  0.74864
log10(SEEM_median)   1.0635     0.3847   2.764  0.00884 **
---
Signif. codes:  0 '***' 0.001 '**' 0.01 '*' 0.05 '.' 0.1 ' ' 1

Residual standard error: 1.948 on 37 degrees of freedom
Multiple R-squared: 0.1712,   Adjusted R-squared: 0.1488
F-statistic: 7.641 on 1 and 37 D; p-value: 0.008842
```

Supplemental References

1. Goldsmith MR, Grulke CM, Brooks RD, Transue TR, Tan YM, Frame A, et al. Development of a consumer product ingredient database for chemical exposure screening and prioritization. *Food Chem Toxicol.* **2014**,65, 269–79. doi: 10.1016/j.fct.2013.12.029

2. Judson R, Richard A, Dix D, Houck K, Elloumi F, Martin M, et al. ACToR--Aggregated Computational Toxicology Resource. *Toxicol Appl Pharmacol.* **2008**, 233, 7–13. doi: 10.1016/j.taap.2007.12.037

3. Isaacs KK, Glen WG, Egeghy P, Goldsmith MR, Smith L, Vallero D, et al. SHEDS-HT: an integrated probabilistic exposure model for prioritizing exposures to chemicals with near-field and dietary sources. *Environ Sci Technol.* **2014,** 48, 12750–12759. doi: 10.1021/es502513w

4. Klepeis NE, Gabel EB, Ott WR, Switzer P. Outdoor air pollution in close proximity to a continuous point source. *Atmos Environ*. **2009**, 43, 3155–3167. doi: 10.1016/j.atmosenv.2009.03.056

5. Mansouri K, Grulke CM, Judson RS, Williams AJ. OPERA models for predicting physicochemical properties and environmental fate endpoints. *J Cheminform*. **2018**, 10. doi: 10.1186/s13321-018-0263-1

6. Nicas M. Estimating exposure intensity in an imperfectly mixed room. *Am Ind Hyg Assoc J.* 1996, 57, 542–550. doi: 10.1080/15428119691014756

7. United States Environmental Protection Agency. Exposure Factors Handbook: 2011 Edition. Washington, DC: National Center for Environmental Assessment; 2011. Report number EPA/600/R-090/052F. Available from: https://www.epa.gov/expobox/about-exposure-factors-handbook.Accessed on 18 January 2022.

8. Wilson R, Jones-Otazo H, Petrovic S, Mitchell I, Bonvalot Y, Williams D, et al. Revisiting Dust and Soil Ingestion Rates Based on Hand-to-Mouth Transfer. *Hum Ecol Risk Assess*. **2013**, 19, 158–188. doi: 10.1080/10807039.2012.685807

9. Deshpande BK, Frey HC, Cao Y, Liu X. Modeling of the Penetration of Ambient PM2.5 to Indoor Residential Microenvironment. Proceedings, 102nd Annual Conference and Exhibition, Air & Waste Management Association; Detroit, MI2009.Paper No. Paper 2009-A-86-AWMA

10. Buist HE, Wit-Bos L, Bouwman T, Vaes WH. Predicting blood:air partition coefficients using basic physicochemical properties. *Regul Toxicol Pharmacol.* **2012**, 62, 23–28. doi: 10.1016/j.yrtph.2011.11.019

11. Weschler CJ, Nazaroff WW. SVOC exposure indoors: fresh look at dermal pathways. *Indoor Air*. **2012**, 22, 356–77. doi: 10.1111/j.1600-0668.2012.00772.x

12. Zartarian VG, Glen G, Smith L, Xue J. SHEDS-Multimedia Model Version 3 (a) Technical Manual; (b) User Guide; and (c) Executable File to Launch SAS Program and Install Model. Agency USEP. Washington, D.C.: U.SA. Environmental Protection Agency; 2008. Report number EPA/600/R-08/118.

13. Linnankoski J, Mäkelä JM, Ranta V-P, Urtti A, Yliperttula M. Computational Prediction of Oral Drug Absorption Based on Absorption Rate Constants in Humans. *J Med Chem*. 2006;49, 3674–3681. doi: 10.1021/jm051231p

14. Sarver JG, White D, Erhardt P, Bachmann K. Estimating Xenobiotic Half-Lives in Humans from Rat Data: Influence of log P. *Environl Heal Pers.* **1997,** 105, 1204–1209. doi: 10.2307/3433898

15. United States Environmental Protection Agency. Consumer Exposure Model (CEM) version 2.1 User Guide. 2019. Available from: https://www.epa.gov/tsca-screening-tools/consumer-exposure-model-cem-version-21-users-guide. Accessed on 18 January 2022.

16. Ozkaynak H, Xue J, Zartarian VG, Glen G, Smith L. Modeled estimates of soil and dust ingestion rates for children. *Risk Anal.* **2011**, 31, 592–608. doi: 10.1111/j.1539-6924.2010.01524.x

17. Nazaroff WW, Weschler CJ. Cleaning products and air fresheners: exposure to primary and secondary air pollutants. *Atmosc Environ.* **2004**, 38, 2841–2865. doi: 10.1016/j.atmosenv.2004.02.040

18. Keil CB, Nicas M. Predicting Room Vapor Concentrations Due to Spills of Organic Solvents. *AIHA* **2003**;64, 445–454. doi: 10.1080/15428110308984838

19. Woodrow JE, Seiber JN, Baker LW. Correlation Techniques for Estimating Pesticide Volatilization Flux and Downwind Concentrations. *Environ Sci & Technol*. 1997, 31, 523–529. doi: 10.1021/es960357w

20. Woodrow JE, Seiber JN, Dary C. Predicting pesticide emissions and downwind concentrations using correlations with estimated vapor pressures. *J Agric Food Chem*. **2001**, 49, 3841–3846. doi: 10.1021/jf010358u

21. Mackay D, van Wesenbeeck I. Correlation of chemical evaporation rate with vapor pressure. *Environ Sci Technol*. **2014**, 48, 10259–10263. doi: 10.1021/es5029074

22. van Wesenbeeck I, Driver J, Ross J. Relationship between the evaporation rate and vapor pressure of moderately and highly volatile chemicals. *Bull Environ Contam Toxicol*. 2008, 80, 315–318. doi: 10.1007/s00128-008-9380-2

23. Howard-Reed C, Corsi RL, Moya J. Mass Transfer of Volatile Organic Compounds from Drinking Water to Indoor Air: The Role of Residential Dishwashers. *Environ Sci & Technol*. 1999, 33, 2266–2272. doi: 10.1021/es981354h

24. McCready D. A Comparison of Screening and Refined Exposure Models for Evaluating Toluene Air Emissions from a Washing Machine. Human and Ecological Risk Assessment: *An International J*. 2012, 19, 972–988. doi: 10.1080/10807039.2012.702596

25. McCready D, Arnold SM, Fontaine DD. Evaluation of Potential Exposure to Formaldehyde Air Emissions from a Washing Machine Using the IAQX Model. Human and Ecological Risk Assessment: *An International J.* 2012, 18, 832–854. doi: 10.1080/10807039.2012.688709

26. Howard C, Corsi RL. Volatilization of chemicals from drinking water to indoor air: the role of residential washing machines. *J Air Waste Manag Assoc*. 1998, 48, 907–914. doi: 10.1080/10473289.1998.10463746

27. Singer BC, Destaillats H, Hodgson AT, Nazaroff WW. Cleaning products and air fresheners: emissions and resulting concentrations of glycol ethers and terpenoids. *Indoor Air*. **2006**, 16, 179–191. doi: 10.1111/j.1600-0668.2005.00414.x

28. Shin HM, McKone TE, Bennett DH. Volatilization of low vapor pressure--volatile organic compounds (LVP-VOCs) during three cleaning products-associated activities: *Pot contri ozone forma. Chemos*. **2016**;153, 130–137. doi: 10.1016/j.chemosphere.2016.02.131

29. Earnest CM. A Two-Zone Model to Predict Inhalation Exposure to Toxic Chemicals in Cleaning Products. Austin, TX: University of Texas Austin; 2009.

30. Zhang Y, Banerjee S, Yang R, Lungu C, Ramachandran G. Bayesian modeling of exposure and airflow using two-zone models. *Ann Occup Hyg.* **2009**, 53, 409–424. doi: 10.1093/annhyg/mep017

31. Nicas M, Plisko MJ, Spencer JW. Estimating benzene exposure at a solvent parts washer. *J Occup Environ Hyg*. **2006**; 3, 284–291. doi: 10.1080/15459620600637390

32. Stanfield Z, Setzer RW, Hull V, Sayre RR, Isaacs KK, Wambaugh JF. Bayesian inference of chemical exposures from NHANES urine biomonitoring data. *JExposure Sci & Environ Epidemiology*. 2022. doi: 10.1038/s41370-022-00459-0
